# Supplementary material for: Self-management challenges following hospital discharge for patients with multimorbidity: a longitudinal qualitative study of a motivational interviewing intervention
Source: BMJ Open. 2021 Jul 30;11(7):e046896. doi: 10.1136/bmjopen-2020-046896 (PMC8327841; doi:10.1136/bmjopen-2020-046896)
Supplement: Supplementary data [file bmjopen-2020-046896supp001.pdf]

Appendix 1_MANUAL

The intervention consists of four cornerstones to ensure a successful transition from the hospital to the home (medication, care plan/follow-up, symptoms and care contacts). The overall objective of the intervention is that the patient gets a secure transition from hospital to home and becomes aware of how they can be more active in self-care.

Cornerstone	Objective
Medication	The patient has knowledge of and justification for which medications they should take and why, and how they should administer/take the medications.
Care plan/ Follow-up	The patient has knowledge and tools for managing follow-up care and treatment, knows why follow-up should take place, where they can turn in case of any problems, and is motivated to manage their own follow-up/care plan.
Symptoms	The patient has knowledge on expected symptoms and signs of deterioration, so they can seek help at the right time and the right level of care (care contacts)
Care contacts	This cornerstone interconnects with the other three cornerstones, i.e., the matter of the patient's care contacts will be relevant in relation to all of them. The objective is that the patient will have knowledge about their care contacts in specialised care and outpatient care, and a method for easily getting in touch during continued care or follow-up of care.

The setup of the conversations:

1. Introduction: Create security, jointly decide on a topic for the conversation.
2. Investigate the patient's views on the matter and assess motivation.
3. Lead into talk about transformation and try to reinforce self-efficacy: Open questions, adapted based on motivation. Scale/rank questions. Information in dialogue. Investigate any ambivalences. Roll with resistance.
4. Closing: Summarize, try to elicit an undertaking.

Conversation 1. Objective: Check how things have been working since the patient got home and make appointments for future conversations.

Conversations 2–5. Objective: The patient and coach work together to identify problems/dysfunctional habits/preconceptions about the disease/treatment, and set an agenda for which change(s) the patient wants to focus on, identify goals with the change, and motivate the patient to start working towards these goals.

Conversation 1:

I wanted to start with checking how things have worked for you since you got home?

The goal of this project is that the two of us will work together to help you feel that you have control over your disease and how you can manage it at home. If it's okay with you, I'd like us to make an appointment for when we can meet (face-to face or by phone) to talk about that a bit more.

Conversations 2–5:

Would you be willing to start with talking a bit about your disease and how it impacts your life? Can you describe to me what you do at home to feel better?

You are describing a lot of important factors that you feel affect your health and how you can take care of yourself. A few other things that I come to think of as important in taking care of your health are medications, being aware of the various symptoms of your disease, your care plan, and your various care contacts. Would you be willing to talk about any of those areas?

MEDICATIONS

Could you show me which of the medications on your list that you take, and tell me a bit about why and how you take them? What do you usually do to get answers to questions you have about medications? Could you describe to me how you feel it works to take the medications on your medication list exactly as the doctor said? Another thing that I was wondering and wanted to ask you about: what do you usually do to keep track of all your medications?

CARE PLAN/FOLLOW-UP

What was decided in regards to your continued care when you were discharged from hospital? What do you usually do when you're waiting for a follow-up? What do you do to keep track of your appointments and follow-ups before you get a notice?

SYMPTOMS

Can you tell me a bit about how your disease makes itself noticeable? What do you do when you get those symptoms? What do you do when you feel that you are getting worse?

CARE CONTACTS

Could you tell me about which care workers you are usually in touch with and why? What do you do when you want to get in touch? What do you usually do to make sure you get the chance to talk about what matters most to you? What do you usually do to remember what the care workers say?
