# Supplementary material for: Self-management challenges following hospital discharge for patients with multimorbidity: a longitudinal qualitative study of a motivational interviewing intervention
Source: BMJ Open. 2021 Jul 30;11(7):e046896. doi: 10.1136/bmjopen-2020-046896 (PMC8327841; doi:10.1136/bmjopen-2020-046896)
Supplement: Author's manuscript [file bmjopen-2020-046896.draft_revisions.pdf]

BMJ Open

BMJ Open is committed to open peer review. As part of this commitment we make the peer review history of every article we publish publicly available.

When an article is published we post the peer reviewers' comments and the authors' responses online. We also post the versions of the paper that were used during peer review. These are the versions that the peer review comments apply to.

The versions of the paper that follow are the versions that were submitted during the peer review process. They are not the versions of record or the final published versions. They should not be cited or distributed as the published version of this manuscript.

BMJ Open is an open access journal and the full, final, typeset and author-corrected version of record of the manuscript is available on our site with no access controls, subscription charges or pay-per-view fees (http://bmjopen.bmj.com).

If you have any questions on BMJ Open's open peer review process please email info.bmjopen@bmj.com

BMJ Open

Self-management challenges following hospital discharge for patients with multimorbidity – a longitudinal qualitative study of a motivational interviewing intervention

Journal:	*BMJ Open*
Manuscript ID	bmjopen-2020-046896
Article Type:	Original research
Date Submitted by the Author:	25-Nov-2020
Complete List of Authors:	Brandberg, Carina; Karolinska Institute, LIME<br>Flink, Maria; Karolinska Institutet, Department of Learning, Informatics, Management and Ethics; Department of Social Work<br>Ekstedt, Mirjam; Karolinska Institutet, LIME; Linneuniversitet,
Keywords:	Heart failure < CARDIOLOGY, Chronic airways disease < THORACIC MEDICINE, PRIMARY CARE, QUALITATIVE RESEARCH

SCHOLARONE™
Manuscripts

I, the Submitting Author has the right to grant and does grant on behalf of all authors of the Work (as defined in the below author licence), an exclusive licence and/or a non-exclusive licence for contributions from authors who are: i) UK Crown employees; ii) where BMJ has agreed a CC-BY licence shall apply, and/or iii) in accordance with the terms applicable for US Federal Government officers or employees acting as part of their official duties; on a worldwide, perpetual, irrevocable, royalty-free basis to BMJ Publishing Group Ltd ("BMJ") its licensees and where the relevant Journal is co-owned by BMJ to the co-owners of the Journal, to publish the Work in this journal and any other BMJ products and to exploit all rights, as set out in our licence.

The Submitting Author accepts and understands that any supply made under these terms is made by BMJ to the Submitting Author unless you are acting as an employee on behalf of your employer or a postgraduate student of an affiliated institution which is paying any applicable article publishing charge ("APC") for Open Access articles. Where the Submitting Author wishes to make the Work available on an Open Access basis (and intends to pay the relevant APC), the terms of reuse of such Open Access shall be governed by a Creative Commons licence – details of these licences and which Creative Commons licence will apply to this Work are set out in our licence referred to above.

Other than as permitted in any relevant BMJ Author's Self Archiving Policies, I confirm this Work has not been accepted for publication elsewhere, is not being considered for publication elsewhere and does not duplicate material already published. I confirm all authors consent to publication of this Work and authorise the granting of this licence.

For peer review only

Self-management challenges following hospital discharge for patients with multimorbidity – a longitudinal qualitative study of a motivational interviewing intervention

Carina Brandberg, Doctoral student [1]

Mirjam Ekstedt, PhD [1,2]*

Maria Flink, PhD [3]

[1]Department of Learning, Informatics, Management and Ethics, LIME, Karolinska Institutet, Stockholm, Sweden

[2]Department of Health and Caring Sciences, Linnaeus University, Kalmar, Sweden

[3]Department of Neurobiology, Care Sciences and Society, Karolinska Institutet, Stockholm, Sweden

*Corresponding author:

Mirjam Ekstedt, PhD

Department of Health and Caring Sciences

Hus Vita, Linnaeus University

SE-391 82 Kalmar, Sweden

Mirjam.ekstedt@lnu.se

Phone: +46 480 446 39

For peer review only

Abstract

Objectives The aim of this study was to explore challenges in self-management activities among people with multimorbidity during a four-week post-discharge period.

Design This is a longitudinal qualitative study using data from a randomized controlled trial (RCT) of motivational interviewing (MI) sessions.

Setting The RCT was conducted at six wards in two hospitals – one university hospital and one general hospital in an urban area, Sweden during 2016–2018.

Participants With a total of 207 participants. Of these, 16 participants from the intervention group, diagnosed with heart failure or chronic obstructive pulmonary disease and at least one other chronic condition, were purposively selected for this study.

Interventions Each participant had four or five post-discharge MI sessions with a trained social worker during a period of approximately four weeks. The sessions were recorded digitally and analyzed using content analysis. Altogether, 70 recorded sessions were analyzed.

Results Two major challenges characterized the post-discharge period in the patients' chain of care, and constituted the longitudinal main category 'A dynamic self-management process after discharge.' These challenges were: 'Managing a system-centered care' and 'Handling the burden of living with multiple illnesses at home post-discharge'.

Conclusions Post-discharge self-management for people with multimorbidity is a process that evolves gradually over time. It requires coordinated person-centered primary healthcare that can meet patients' needs, wherever they are along the care trajectory and when they need it most.

For peer review only - http://bmjopen.bmj.com/site/about/guidelines.xhtml

Trial registration number: Trial Registration Number: NCT02823795, date of registration 2016-07-05.

Keyword: Self-management, patient- centered care, multimorbidity, patient transfer, Motivational Interviewing, qualitative research

Strengths and limitations of this study

- The longitudinal design with repeated sessions between patients and social workers ensure large amounts of high-quality data.
- The patients were selected, after the randomization process, to achieve as rich a variety of data as possible, by including different genders, ages, coaches, and hospital settings.
- A small number of participants (n 16); however, saturation was achieved and the repeated sessions included 70 recorded and analyzed sessions.
- All patients came from same geographical area in Sweden, which is a limitation regarding transferability.

For peer review only

Introduction

In Sweden, about 25% of the population have multimorbidity, and this group accounts for 50% of the total healthcare costs (1). There are many definitions of multimorbidity (2), but one frequently used in literature – and in this study – is the co-existence of two or more chronic conditions, where all diagnoses can be of equal importance (3, 4). Healthcare systems all around the world are organized around single diseases (4-7) and most clinical practice guidelines focus on the management of a single disease (8). Such fragmentation of services causes a large number of negative consequences, such as medical errors and misdiagnosis (9). It is therefore not surprising that patients with multimorbidity encounter challenges in the healthcare system, or that patients are especially vulnerable during care transitions.

Discharge from hospital to home is a challenging care transition (10), with known obstacles in the form of adverse events (11, 12), re-hospitalization (13), and new or worsening symptoms (14). A crucial factor for effective care transition to home is support of patient self-management, preferably initiated at the hospital; studies indicate a beneficial effect on the risk for 30-day readmission (15, 16). Self-management is "an individual's ability to manage the symptoms, treatment, physical and psychosocial consequences and life style changes inherent in living with a chronic condition" (17). A patient's capacity for self-management seems to be a crucial factor in care transitions, as high levels of patient activation have been associated with decreased healthcare usage (18). Self-management support during care transitions is especially important, as patients often feel that the discharge encounter does not prepare them for post-hospitalization self-management and that no one takes overall responsibility for the coordination of their care (19). There is an

For peer review only - http://bmjopen.bmj.com/site/about/guidelines.xhtml

international consensus that the healthcare system needs to be reorganized to provide continuity, coordination, and – most importantly – a patient-centered focus that supports self-management (19), adapted to each patient's preferences and goals (6). People with multimorbidity are a population in true need of patient-centered self-management support, taking their preferences and needs into account (20).

Challenges to and facilitators of self-management have been extensively explored, but self-management over time is scarcely described (21). There are even fewer descriptions of how challenges to self-management are experienced and handled during the most vulnerable period, i.e., the first weeks after a hospital discharge – to our knowledge there are none. This study aimed to explore the process of and challenges to self-management activities, as expressed by patients with multimorbidity in a 4-week post-discharge motivational interviewing (MI) consultation trial.

Materials and Methods

Study design

This is a longitudinal qualitative study of self-management after hospitalization in patients with multimorbidity, based on four to five MI sessions per patient, in a randomized controlled trial (RCT). For details of the RCT, see the study protocol (22). A pilot study with three participants was performed before the start of the study, to test the MI method.

The consolidated criteria for reporting qualitative research (COREQ) were used for explicit and comprehensive reporting (23).

For peer review only - http://bmjopen.bmj.com/site/about/guidelines.xhtml

The MI intervention

The MI is grounded in self-determination theory, a macro-theory of human motivation. In this study, MI was used to increase patient activation in self-management through enhancing a person's autonomy, competence, and relatedness (20). When people become more motivated, engaged, and experience more psychological well-being, this predicts positive health behavior (24). Three specific components of autonomy support are suggested: taking the perspective of the patient, providing the patient with choices, and providing a rationale when choices are not possible (25, 26). The principle of autonomy support is at the heart of the clinical approach called MI (27), which can be delivered in a single session or through multiple sessions (28).

The patients in the intervention group each had four to five MI sessions with one of three trained hospital social worker during a period of approximately four weeks. The first session was conducted 1–2 days after discharge, to establish a first contact and capture any questions that arose early post-discharge. The following sessions were conducted 1, 2, 3, and 4 weeks post-hospitalization. After the fourth session, some patients expressed that they did not need any more support, why the coaching ended after four sessions. The MI sessions were held either over the phone or face-to-face in clinic, depending on what the patient preferred. The length of the MI sessions varied between 10 and 90 minutes. The first session was usually the shortest, due to fatigue and having a lot of information to absorb post-discharge. All sessions were audio-recorded with the permission of the patients.

All three hospital social workers had MI training and received MI guidance from a psychologist, a member of MINT (Motivational Interviewing Network of Trainers), once a month during the trial. The core of the MI sessions was to motivate and empower the

For peer review only - http://bmjopen.bmj.com/site/about/guidelines.xhtml

patient to take active part in self-management regarding four important aspects and to create goals for these aspects: 1) managing medications, 2) managing symptoms and/or signs of worsening illness after discharge, 3) acquiring knowledge of follow-up, and 4) acquiring knowledge of and control over whom to contact for different healthcare needs (22). The patients were also encouraged to discuss other aspects of their self-management that were important to them. The hospital social workers had no prior relationship with the patients.

Selection of participants and data

Recruitment for the MI intervention (22) was conducted at six wards in two hospitals: one university hospital and one general hospital in an urban area, Sweden. Data were collected from 2016-08-24 to 2018-05-17. The participants were recruited to the RCT during hospitalization for any cause, if they fulfilled the following inclusion criteria: diagnosed with chronic heart failure (CHF) or/and chronic obstructive pulmonary disease (COPD), able to speak and understand enough Swedish to participate in the MI sessions, not diagnosed with dementia or mild cognitive impairment, and not having a non-resuscitate statement in their medical record. Patients received both written and verbal information about the study and the authors, with the possibility to discuss any questions they might have. Those who needed time to decide were offered that until discharge.

From a total of 207 patients in the RCT, 16 patients from the intervention group (n = 104) were purposively selected, to include participants with at least two chronic conditions and a variety of diagnoses, ages, genders, and hospital settings. The patients were also selected to cover MI sessions held by all three coaching social workers and throughout the intervention period. An overview of the patients' characteristics can be found in Table 1. The MI sessions,

For peer review only - http://bmjopen.bmj.com/site/about/guidelines.xhtml

i.e., conversations between the patients and the social workers, were audio-recorded and transcribed verbatim. Transcribed data consisted of four to five MI sessions per patient: 70 recorded sessions in all. To ensure that optimal sample size and saturation had been achieved, two more series of sessions were analyzed (29). No participants dropped out.

Table 1. Characteristics of the participants (N = **16**).

Characteristics	Number (SD)
**Age, mean**	71.0 (10.0)
Male, mean	69.3 (11.0)
Female, mean	72.1 (9.5)
**Gender**	
Female	7
**Place of birth**	
In Sweden	12
**Marital status**	
Married	7
Single/widowed/separated	9
**Closest next-of-kin**	
Relative[1]	14
No next-of-kin	2
**Education level**	
No education	1
Elementary school	5
High school/upper secondary school	7
College/university	3
**Income (Swedish krona/month)**	
< 10,000	1

For peer review only

10,000–20,000	6
20,000–50,000	7
> 50,000	1
**Specific diagnosis[2]**	
Congestive heart failure	9
Chronic obstructive pulmonary diseases	9
Hypertension	7
Diabetes	6
Renal failure	3
Anemia	3
**Chronic conditions per individual (N)**	
2–4	2
5	6
6	4
7	4
**Diagnoses (N), males, mean**	6.0 (1.0)
**Diagnoses (N), females, mean**	5.0 (1.4)
**Charlson comorbidity index, age-adjusted, mean**	6.7 (2.5)

[1]Husband/wife/partner, child, or friend.

[2]Data taken from electronic/medical records.

Analysis

The transcribed sessions were analyzed using inductive qualitative content analysis, including open coding and creating categories through abstraction (30). First, the full sequence of the four to five MI sessions for each patient was listened to, and the transcripts

For peer review only - http://bmjopen.bmj.com/site/about/guidelines.xhtml

were read as a whole, to achieve a comprehensive understanding of the experience of self-management after discharge. Then, units of meaning were selected, condensed, and coded for one participant at a time, starting from the first session and continuing in chronological order (31). First, each set of sessions was abstracted and formulated to sub-categories separately, for all patients, i.e., all the first sessions, then all the second sessions and so on. The session- specific sub-categories were thereafter grouped into 14 sub-generic categories, and these were grouped into two generic categories. Next, a latent, longitudinal analysis was conducted across the five sessions with a focus on changes in the self-management process over time. This longitudinal analysis identified a main category across the generic categories, 'dynamic self-management process after discharge.' An example of the analytical steps from units of meaning to main category can be found in table 2.

Table 2. An example of the analytical steps from units of meaning to main category (ID 16).

Unit of meaning	Heading (Code)	Sub-category	Sub-generic category	Generic category	Main category
Mm…and with home care and other help, you´re never really free	Need for home care decreases sense of freedom	Need for independence delays care seeking	Personal experiences direct care seeking behavior	'Handling the burden of living with multiple illnesses post-discharge.'	'Dynamic self-management process after discharge'

CB coded all data. The coding and interpretation processes were conducted in continuous discussions between the three authors (CB, MF, and ME). Excel was used to sort codes and categories. CB and ME are registered nurses, and MF is social worker. MF and ME are well experienced in qualitative research.

For peer review only - http://bmjopen.bmj.com/site/about/guidelines.xhtml

Patient and public involvement

Patients were not involved in the design or analysis of this study, due to this population's multimorbidity and high mean age. However, the used method (MI) in this study is patient centred, which mean that the patients are free to express and participate to their extent. Patients were offered to take part of the result and those who are interested will receive the published article including a plain language summary.

Result

Our data showed that patients with multimorbidity struggled with self-management in the first four weeks at home post-discharge. The longitudinal main category identified, 'A dynamic self-management process after discharge,' consisted of two generic categories that characterized this transitional period in a patient's chain of care: 'Managing a system-centered care' and 'Handling the burden of living with multiple illnesses post-discharge.'

A dynamic self-management process after discharge

The longitudinal analysis showed that self-management after hospital discharge was a dynamic process with several shifting features that evolved gradually over time. Difficulties that appeared during the first week were replaced by new obstacles in the second or third week, i.e., the challenges shifted as the patients recovered and gradually returned to normal life. During the first week post-discharge, the first two sessions concerned how the patients adjusted to their changed health conditions and the patients had many questions, especially regarding medication management. Some of the most salient challenges were fatigue, symptoms impairing activities in daily life and causing social isolation, and handling of new

For peer review only - http://bmjopen.bmj.com/site/about/guidelines.xhtml

For peer review only

medical regimens. The post-discharge period was also characterized by a struggle to understand the new situation, including understanding information from the hospital.

As fatigue gradually declined, a new topic of health-related anxiety and stress was raised during the second week post-discharge (i.e., the third session). For example, anxiety and stress related to feelings of being a burden to relatives, neighbors, or colleagues, being unsure which healthcare contact was responsible for care post-discharge, or not receiving sick pay from the Swedish Social Insurance Agency. Questions regarding management of stress-related problems dominated the following sessions and peaked during the fourth week. A reduction of fatigue and other symptoms impairing daily life could be seen at the last session.

Managing a system-centered care

The patients described the period following hospitalization as not being centered around their needs, but being based on the design of the healthcare system. The patient had to adapt to the system, rather than the system being aligned to their needs as a patient with multimorbidity. “I have a cardiologist and I have a pulmonologist and I have a general practitioner then, and these three never meet.” (ID 20)

Different physicians used different treatment strategies and several of the patients felt that new GPs lacked knowledge about them and their clinical picture. This could result in insufficient information on diagnosis and treatment, when to take medicines, and possible side effects of treatment. “Some doctors do not want to prescribe my medications. Medications that I should have, that I have taken for a long time.” (ID 4) Patients in this study also described difficulties with getting in touch with their GPs, sometimes resulting in prolonged improper medical treatment. “… then I have to keep track of this doctor at the

For peer review only - http://bmjopen.bmj.com/site/about/guidelines.xhtml

healthcare center and talk to him during his phone hours, as he has 30 minutes two days a week and so he has no phone hours today.” (ID 25)

High turnover of GPs, shortage of time in consultations, and neglect of the patient perspective contributed to non-trusting relationships between patients and GPs. The high turnover of GPs affected patients’ continuity of care. “I have been assigned to him, I have not chosen a doctor myself and everything feels very uncertain. You have to start all over again and that is really hard.” (ID 51)

For patients with newly diagnosed heart failure, the follow-up after discharge was conducted at a heart failure reception, where each patient got further information, the possibility to ask questions, and, if needed, evaluation and adjustment of medicines. Despite this, patients lacked an opportunity to discuss their medications with a physician, instead of having a specialist nurse evaluating the physician’s prescriptions. In this way, the heart failure reception was perceived to add to the fragmentation through involving yet another healthcare contact. In one case, a patient showed creativity by making an Excel file organizing all his 21 healthcare contacts by color, based on what diagnosis they provided care for. During the MI sessions, strategies on how to prepare for healthcare encounters to overcome the lack of continuity were addressed, for example by discussing important topics to mention at meetings.

Another challenge was that medical record systems differed between departments, forcing patients to take responsibility for gathering information on their own care from their various caregivers. One man said (ID 50): “The healthcare system is made for only one diagnosis at a time.” Several participants solved this issue by always carrying their most current patient record and list of medications with them.

For peer review only - http://bmjopen.bmj.com/site/about/guidelines.xhtml

Another issue was when the choice of medicine was ruled by price rather than to simplify drug management for the patient. A woman said (ID 69): “They (pharmacy) want to give me Becotide because it is cheaper at the pharmacy, but my heart does not like Becotide and my lung doctor has said ‘You must not change it.’ And they (pharmacy) say, ‘But it is more expensive,’ but I’m paying for it.” For some patients, the national pharmaceutical policy of offering the cheapest products complicated drug management, as they found it difficult to understand and remember the different generic names. Another barrier regarding medicines was incomplete lists of medicines, especially in the case of temporary medicines, which are often missing from such lists.

Handling the burden of living with multiple illnesses at home post-discharge

Lack of energy and strength in the first weeks after discharge affected the patients’ ability to understand information on how to manage new symptoms, and to make the necessary adjustments in daily life. A man said (ID 36): “but I'm a patient, I'm not a normal person, I’m a patient, I’m not functioning.”

Fatigue and impaired physical functioning also led to the patients feeling socially isolated in their homes. For example, having to take just a few steps on stairs with impaired balance, which was a challenge already under ordinary conditions, now significantly affected daily life. In one case, a woman’s fear of sudden dizziness and a risk of falling on the stairs led her to repeatedly cancel appointments with her general practitioner (GP). Some of the patients also expressed uncertainty and had a lot of questions when they got home. “…if you have undergone surgery or something at hospital, you receive this note and the doctor rambles on a lot but then, when you get home, you wonder what they actually said.” (ID 25)

For peer review only - http://bmjopen.bmj.com/site/about/guidelines.xhtml

Self-management also required high level of attentiveness to subtle symptoms in order to identify diagnoses correctly and respond properly. Some patients expressed concerns regarding their ability to identify which disease their symptoms derived from. For example, a patient with COPD, CHF, and anemia (ID 20) misinterpreted her breathing problems as related to her COPD and thus acted on the symptoms “as usual.” She said: “Yes, you get used to feeling bad.” This delayed her from seeking care, which led to hospitalization for what turned out to be serious anemia. Some patients also expressed difficulties in identifying possible side effects when prescribed several new medicines at once. Some found their own ways of managing their daily medication, for example by delegating this responsibility to relatives.

An acute hospital stay was experienced as a setback, which reduced strength and affected motivation for self-management. It was perceived as having to start all over again, to regain strength and independence in daily life. Keeping physical activity levels up was regarded as especially stressful after a stay in hospital. “I had a walk…or treadmill for a long time, but I haven’t been able to walk on it, since I’ve been in such bad shape, but now… And then there’s so much else going on around me and…” (ID 7) Most patients were aware of the importance of physical activity for managing daily life. Challenges to exercise included health limitations, repeated infections, surgery, lack of company, and inclement weather.

The strong desire for autonomy was an asset in the handling of symptoms and challenges post-discharge. In contrast, adjusting to being dependent on home healthcare or informal care, due to increased fatigue or impaired health, was hard for some. Some participants also described having felt shame since childhood when asking for help, and taking pride in caring for themselves, which was strongly associated with their self-image. One woman said: “I

For peer review only - http://bmjopen.bmj.com/site/about/guidelines.xhtml

want to take care of myself as well, that's the main thing I do." (ID 51) Such striving for independence could be a health risk due to seeking care too late or refusing treatment such as dialysis because of fears of becoming dependent.

In some cases, a care relationship with a relative or friend may have delayed getting adequate help. In one case, a very attentive neighbor provided food and care for a woman who could not get out of her couch after three serious falls and multiple fractures, which delayed the hospitalization by four weeks. In another case, a patient was cared for at home by her husband for several weeks before she went to hospital, and only then did she realize that she had serious anemia. Lacking the motivation to book an appointment with primary care was also related to having a poor relationship with or lacking trust in the GP; some waited for so long that they had to seek emergency care instead.

Discussion

The result of this analysis of a series of MI sessions during four weeks post-hospitalization showed that self-management after discharge is a dynamic process that is affected by managing a system-centered care and the burden of living with multiple illnesses. Overall, patients with multimorbidity need support during the first couple of weeks post-discharge, implying that they are sensitive to a system-centered healthcare system.

The period immediately after discharge from hospital is a vulnerable one, with increased risk of new hospitalizations. Patients with multimorbidity may be especially vulnerable in the management of their chronic illnesses. Our results, in line with previous research (24, 32), showed that self-management is a dynamic process that changes rapidly during the first weeks post-discharge. The patients experienced shifting needs and struggled with self-management in a system that was designed for a single-disease population, not one of

For peer review only - http://bmjopen.bmj.com/site/about/guidelines.xhtml

increasing age and with an increasing number of diseases. Thus, the patients experienced a triple whammy, as they had to handle the burden of their illness, including fatigue, the burden of self-management of multiple diagnoses, and the burden of being in a system not designed to meet their needs (5-7, 33). In this, the patients experienced that they were left alone to handle their self-management tasks, which especially affected those who were unsure about their own abilities (34). The patients' experiences of a system that did not meet their needs could be reinforced by the design of the Swedish healthcare system, which has one of the shortest lengths of hospital stays in the EU (5.9 days) and the third lowest percentage of general practitioners among countries in the OECD (35). This affects this group of patients, due to a need for prompt follow-up within primary healthcare post-discharge.

The current study also highlights that patients cannot cope with more activities than what is "mandatory" after discharge, as they struggle with self-management while being fatigued. A large proportion of those invited to participate in the RCT in this study declined to participate due to fatigue and severe symptoms (36), indicating that patients with complex care needs might need support for more than just self-management during the post discharge period. The system-centered healthcare system was a sub-optimal condition for motivation and self-management of illness post-discharge. The population in this study was in a great need of healthcare that could provide them with a patient- and family-centered approach, as suggested by other studies (3, 37). Initiatives in this direction are seen within a national healthcare reform (38). However, understanding how to provide this type of care efficiently is an enormous challenge (3).

For peer review only

For peer review only - http://bmjopen.bmj.com/site/about/guidelines.xhtml

Strengths and limitations

A strength of this study is the longitudinal design with four to five MI sessions, conducted during a four-week post-discharge period. However, there is a need to consider that the audio-recorded MI sessions probably affected the post-discharge period in several ways, as they aimed to increase patients' motivation for self-management. The longitudinal main category "dynamic self-management process after discharge" was considered to capture the patients' transition processes and their experiences of how self-management and recovery changed during the first weeks post-discharge. However, this process might have been different for persons with multimorbidity who did not receive MI sessions.

The MI sessions were directed at four aspects relevant to post-discharge self-management: (1) medication, 2) symptoms, 3) follow-up, and 4) whom to contact for different healthcare needs. However, in accordance with the MI methodology, patients were also encouraged to discuss any relevant problems that occurred post-discharge, meaning that the sessions were guided by patient needs. The results indicated that the patients felt free to discuss several different aspects, both the four mentioned above and others. The MI coaches were hospital social workers, i.e., not medically trained staff, which could have led to the sessions focusing on more general aspects of self-management and not specifically on medical aspects.

To ensure trustworthiness (29), researchers' reflections were continuously written down during the analysis. These reflections were used in the analysis process. The analysis was repeatedly discussed between the three authors and within the research group. Examples of quotations from the interviews are presented in the findings. The patients were selected to achieve as rich a variety of data as possible, by including different genders, ages, coaches, and hospital settings. We did not return the results to the participants for verification, as we

For peer review only - http://bmjopen.bmj.com/site/about/guidelines.xhtml

considered that this group of old, sick patients might not be able to assess the correctness of the analysis (29).

The data collection ensured large amounts of high-quality data, thanks to the repeated sessions between patients and social workers. These sessions contributed to building trust between patients and coaches, which probably made it more likely that rich information was obtained and that any misunderstandings or distortions were uncovered (39).

Conclusion

Managing self-management post-discharge, in a population with multimorbidity, is a process that evolves gradually over time, from extensive fatigue to emergent feelings of anxiety and stress over self-management activities. To be able to meet patient needs, prompt and coordinated patient-centered primary care is highly warranted.

For peer review only

For peer review only - http://bmjopen.bmj.com/site/about/guidelines.xhtml

Declarations

Ethics approval and consent to participate

The study was approved by the Regional Research Ethics Committee (No. 2014/149831/2) and was performed in line with the Helsinki Declaration (2014). Patients who met the inclusion criteria and agreed to participate provided written informed consent.

Consent for publication

Consent for recording the MI sessions and publication was acquired during the inclusion of the participants.

Availability of data and materials

The datasets used during the current study are available from the corresponding author on request.

Competing interests

The authors declare that they have no competing interests.

Acknowledgements

The authors thank all the patients for their participation in this study. The authors would also like to thank Marita Renman-Bengtcén, social worker, Camilla Lundström, social worker, and Camilla Kjellberg, social worker, for performing all the MI coaching sessions with the patients, and Johan Holmberg, psychologist and member of MINT, and Sara Hammer, psychologist and member of MINT, for mentoring and providing insightful coaching to the coaches.

For peer review only - http://bmjopen.bmj.com/site/about/guidelines.xhtml
For peer review only

Funding

Funding was received from the Doctoral School in Health Care Sciences (FiV) [#4-3084/2014], Karolinska Institutet, FORTE [#2015-00412], and Vårdalstiftelsen [#2014-0026]. The support from FiV and Vårdalsstiftelsen was unconditional. The data collection, design, analysis, interpretation, and reporting were performed without their interference.

Authors' contributions

ME and MF contributed with the conception and design of the study and the obtaining of funding. CB and MF performed inclusion of all participants in the RCT. CB conducted the initial coding of transcribed data in discussion with ME and MF and then performed a longitudinal analysis with a focus on changes in the self-management process over time. In discussions with CB, MF and ME contributed to the abstraction, interpretation of codes, and formulation of sub-categories and main categories. CB drafted the manuscript. MF and ME contributed with substantial contents to the writing. All authors read and approved the final manuscript prior to submission.

For peer review only

References

1. Vårdanalys M. Vårdval och vårdutnyttjande–Hur fördelas vården mellan olika socioekonomiska grupper vid vårdval i specialistvården. Myndigheten för Vårdanalys, Stockholm[Google Scholar]. 2014.
2. Fratiglioni L, Marengoni A, Meinow B, Karp A. Multipla hälsoproblem bland personer över 60 år - En systematisk litteraturöversikt om förekomst, konsekvenser och vård. Regeringskansliet. Statens Offentliga Utredningar. SOU 2010:48.; 2010.
3. Boyd CM, Fortin M. Future of multimorbidity research: how should understanding of multimorbidity inform health system design?(Report). Public Health Reviews. 2010;32(2):451.
4. Violan C, Foguet-Boreu Q, Flores-Mateo G, Salisbury C, Blom J, Freitag M, et al. Prevalence, Determinants and Patterns of Multimorbidity in Primary Care: A Systematic Review of Observational Studies. PloS one. 2014;9(7):e102149.
5. O'Hare AM, Rodriguez RA, Bowling CB. Caring for patients with kidney disease: shifting the paradigm from evidence- based medicine to patient- centered care. Nephrology Dialysis Transplantation. 2016;31(3):368-75.
6. Salisbury C, Man M-S, Bower P, Guthrie B, Chaplin K, Gaunt DM, et al. Management of multimorbidity using a patient-centred care model: a pragmatic cluster-randomised trial of the 3D approach. The Lancet. 2018;392(10141):41-50.
7. Fratiglioni L, Marengoni A, Meinow B, Karp A. Multipla hälsoproblem bland personer över 60 år.
8. American Geriatrics Society Expert Panel on the Care of Older Adults With M. Patient-Centered Care for Older Adults with Multiple Chronic Conditions: A Stepwise Approach from the American Geriatrics Society. Journal of the American Geriatrics Society. 2012;60(10):1957-68.
9. Kern L, Safford M, Slavin M, Makovkina E, Fudl A, Carrillo J, et al. Patients' and Providers' Views on Causes and Consequences of Healthcare Fragmentation in the Ambulatory Setting: a Qualitative Study. Journal of General Internal Medicine. 2019;34(6):899-907.
10. Organization WH. Transitions of Care: Technical Series on Safer Primary Care 2016. Available from: https://apps.who.int/iris/bitstream/handle/10665/252272/9789241511599-eng.pdf?sequence=1.
11. Forster AJ, Murff HJ, Peterson JF, Gandhi TK, Bates DW. The incidence and severity of adverse events affecting patients after discharge from hospital.(Abstract). Annals of Internal Medicine. 2003;138(3):161.
12. Kripalani S, Lefevre F, Phillips CO, Williams MV, Basaviah P, Baker DW. Deficits in Communication and Information Transfer Between Hospital-Based and Primary Care Physicians: Implications for Patient Safety and Continuity of Care. JAMA. 2007;297(8):831-41.
13. Vashi AA, Fox JP, Carr BG, D'onofrio G, Pines JM, Ross JS, et al. Use of Hospital-Based Acute Care Among Patients Recently Discharged From the Hospital. JAMA. 2013;309(4):364-71.
14. Epstein K, Juarez E, Loya K, Gorman MJ, Singer A. Frequency of new or worsening symptoms in the posthospitalization period. Journal of hospital medicine. 2007;2(2):58.
15. Leppin AL, Gionfriddo MR, Kessler M, Brito JP, Mair FS, Gallacher K, et al. Preventing 30-Day Hospital Readmissions: A Systematic Review and Meta-analysis of Randomized Trials. JAMA Internal Medicine. 2014;174(7):1095-107.
16. Braet A, Weltens C, Sermeus W. Effectiveness of discharge interventions from hospital to home on hospital readmissions: a systematic review. JBI database of systematic reviews and implementation reports. 2016;14(2):106.
17. Barlow J, Wright C, Sheasby J, Turner A, Hainsworth J. Self-management approaches for people with chronic conditions: a review. Patient Education and Counseling. 2002;48(2):177-87.
18. Kinney RL, Lemon SC, Person SD, Pagoto SL, Saczynski JS. The association between patient activation and medication adherence, hospitalization, and emergency room utilization in

For peer review only - http://bmjopen.bmj.com/site/about/guidelines.xhtml

patients with chronic illnesses: A systematic review. Patient Education and Counseling. 2015;98(5):545-52.

19. Flink M, Ekstedt M. Planning for the Discharge, not for Patient Self-Management at Home – An Observational and Interview Study of Hospital Discharge. International Journal of Integrated Care. 2017;17(6).

20. Deci EL, Ryan RM. Self-determination theory in health care and its relations to motivational interviewing: a few comments.(Commentary)(Report). The International Journal of Behavioral Nutrition and Physical Activity. 2012;9:24.

21. Audulv Å. The over time development of chronic illness self-management patterns: a longitudinal qualitative study. BMC public health. 2013;13(1):452.

22. Flink M, Lindblad M, Frykholm O, Kneck Å, Nilsen P, Årestedt K, et al. The Supporting Patient Activation in Transition to Home (sPATH) intervention: a study protocol of a randomised controlled trial using motivational interviewing to decrease re-hospitalisation for patients with COPD or heart failure. BMJ Open. 2017;7(7).

23. Tong A, Sainsbury P, Craig J. Consolidated criteria for reporting qualitative research (COREQ): a 32-item checklist for interviews and focus groups. International journal for quality in health care. 2007;19(6):349-57.

24. Johan YYN, Nikos N, Cecilie T-N, Edward LD, Richard MR, Joan LD, et al. Self-Determination Theory Applied to Health Contexts: A Meta-Analysis. Perspectives on psychological science. 2012;7(4):325-40.

25. Rollnick S. Motiverande samtal i hälso- och sjukvård : att hjälpa människor att ändra beteende. 1. uppl. ed. Miller WR, Butler CC, editors. Lund : Studentlitteratur; 2009.

26. Sheldon KM. Self-determination theory in the clinic: motivating physical and mental health. Williams G, Joiner TE, editors. New Haven, Conn. ; London : Yale University Press; 2003.

27. Miller WR, Rollnick S. Meeting in the middle: motivational interviewing and self-determination theory. The international journal of behavioral nutrition and physical activity. 2012;9(1):25-.

28. Lundahl B, Moleni T, Burke BL, Butters R, Tollefson D, Butler C, et al. Motivational interviewing in medical care settings: A systematic review and meta-analysis of randomized controlled trials. Patient education and counseling. 2013;93(2):157-68.

29. Elo S, Kääriäinen M, Kanste O, Pölkki T, Utriainen K, Kyngäs H. Qualitative Content Analysis: A Focus on Trustworthiness. SAGE Open. 2014;4(1):215824401452263.

30. Elo S, Kyngäs H. The qualitative content analysis process. Journal of Advanced Nursing. 2008;62(1):107-15.

31. Saldana, Johnny. Longitudinal qualitative research:Analyzing change through time. Walnut Creek, CA: Altamira Press; 2003.

32. Ko D, Bratzke LC, Roberts T. Self-management assessment in multiple chronic conditions: A narrative review of literature. International Journal of Nursing Studies. 2018;83:83-90.

33. Clarfield A, Bergman H, Kane R. Fragmentation of care for frail older people--An international problem. Experience from three countries: Israel, Canada, and the United States. Journal of the American Geriatrics Society. 2001;49(12):1714-21.

34. Audulv Å, Norbergh KG, Asplund K, Hörnsten Å. An ongoing process of inner negotiation – a Grounded Theory study of self-management among people living with chronic illness. Journal of Nursing and Healthcare of Chronic Illness. 2009;1(4):283-93.

35. Oecd, European Observatory on Health Systems and P. Sweden: Country Health Profile 2017. Brussels: OECD Publishing; 2017.

36. Flink M, Brandberg C, Ekstedt M. Why patients decline participation in an intervention to reduce re-hospitalization through patient activation: whom are we missing?(Report). Trials. 2019;20(1).

37. Ploeg J, Canesi M, D Fraser K, McAiney C, Kaasalainen S, Markle-Reid M, et al. Experiences of community-dwelling older adults living with multiple chronic conditions: a qualitative study. BMJ Open. 2019;9(3):e023345.

For peer review only - http://bmjopen.bmj.com/site/about/guidelines.xhtml

38. Anna Nergårdh LA, Anna-Klara Brage, Niklas Eklöf, Caroline-Olivia Elgán, Max Printz. God och nära vård-En reform för ett hållbart hälso- och sjukvårdssystem. In: Regeringskansliet, Utredningar SO, editors. 2020. p. 492.
39. Polit DF. Nursing research : generating and assessing evidence for nursing practice. Philadelphia : Wolters Kluwer Health/Lippincott Williams & Wilkins; 2012.

For peer review only

For peer review only - http://bmjopen.bmj.com/site/about/guidelines.xhtml

COREQ (COnsolidated criteria for REporting Qualitative research) Checklist

A checklist of items that should be included in reports of qualitative research. You must report the page number in your manuscript where you consider each of the items listed in this checklist. If you have not included this information, either revise your manuscript accordingly before submitting or note N/A.

Topic	Item No.	Guide Questions/Description	Reported on Page No.
**Domain 1: Research team and reflexivity**			
Personal characteristics			
Interviewer/facilitator	1	Which author/s conducted the interview or focus group?	
Credentials	2	What were the researcher's credentials? E.g. PhD, MD	
Occupation	3	What was their occupation at the time of the study?	
Gender	4	Was the researcher male or female?	
Experience and training	5	What experience or training did the researcher have?	
Relationship with participants			
Relationship established	6	Was a relationship established prior to study commencement?	
Participant knowledge of the interviewer	7	What did the participants know about the researcher? e.g. personal goals, reasons for doing the research	
Interviewer characteristics	8	What characteristics were reported about the inter viewer/facilitator? e.g. Bias, assumptions, reasons and interests in the research topic	
**Domain 2: Study design**			
Theoretical framework			
Methodological orientation and Theory	9	What methodological orientation was stated to underpin the study? e.g. grounded theory, discourse analysis, ethnography, phenomenology, content analysis	
Participant selection			
Sampling	10	How were participants selected? e.g. purposive, convenience, consecutive, snowball	
Method of approach	11	How were participants approached? e.g. face-to-face, telephone, mail, email	
Sample size	12	How many participants were in the study?	
Non-participation	13	How many people refused to participate or dropped out? Reasons?	
Setting			
Setting of data collection	14	Where was the data collected? e.g. home, clinic, workplace	
Presence of non-participants	15	Was anyone else present besides the participants and researchers?	
Description of sample	16	What are the important characteristics of the sample? e.g. demographic data, date	
Data collection			
Interview guide	17	Were questions, prompts, guides provided by the authors? Was it pilot tested?	
Repeat interviews	18	Were repeat inter views carried out? If yes, how many?	
Audio/visual recording	19	Did the research use audio or visual recording to collect the data?	
Field notes	20	Were field notes made during and/or after the inter view or focus group?	
Duration	21	What was the duration of the inter views or focus group?	
Data saturation	22	Was data saturation discussed?	
Transcripts returned	23	Were transcripts returned to participants for comment and/or	

For peer review only

Topic	Item No.	Guide Questions/Description	Reported on Page No.
		correction?	
**Domain 3: analysis and findings**			
Data analysis			
Number of data coders	24	How many data coders coded the data?	
Description of the coding tree	25	Did authors provide a description of the coding tree?	
Derivation of themes	26	Were themes identified in advance or derived from the data?	
Software	27	What software, if applicable, was used to manage the data?	
Participant checking	28	Did participants provide feedback on the findings?	
Reporting			
Quotations presented	29	Were participant quotations presented to illustrate the themes/findings? Was each quotation identified? e.g. participant number	
Data and findings consistent	30	Was there consistency between the data presented and the findings?	
Clarity of major themes	31	Were major themes clearly presented in the findings?	
Clarity of minor themes	32	Is there a description of diverse cases or discussion of minor themes?	

Developed from: Tong A, Sainsbury P, Craig J. Consolidated criteria for reporting qualitative research (COREQ): a 32-item checklist for interviews and focus groups. *International Journal for Quality in Health Care*. 2007. Volume 19, Number 6: pp. 349 – 357

Once you have completed this checklist, please save a copy and upload it as part of your submission. DO NOT include this checklist as part of the main manuscript document. It must be uploaded as a separate file.

For peer review only

BMJ Open

Self-management challenges following hospital discharge for patients with multimorbidity – a longitudinal qualitative study of a motivational interviewing intervention

Journal:	*BMJ Open*
Manuscript ID	bmjopen-2020-046896.R1
Article Type:	Original research
Date Submitted by the Author:	24-Jun-2021
Complete List of Authors:	Brandberg, Carina; Karolinska Institute, LIME<br>Ekstedt, Mirjam; Karolinska Institutet, LIME; Linneuniversitet,<br>Flink, Maria; Karolinska Institutet, Department of Neurobiology, Care Sciences and Society; Department of Social Work
**Primary Subject Heading**:	Qualitative research
Secondary Subject Heading:	Nursing
Keywords:	Heart failure < CARDIOLOGY, Chronic airways disease < THORACIC MEDICINE, PRIMARY CARE, QUALITATIVE RESEARCH

SCHOLARONE™
Manuscripts

I, the Submitting Author has the right to grant and does grant on behalf of all authors of the Work (as defined in the below author licence), an exclusive licence and/or a non-exclusive licence for contributions from authors who are: i) UK Crown employees; ii) where BMJ has agreed a CC-BY licence shall apply, and/or iii) in accordance with the terms applicable for US Federal Government officers or employees acting as part of their official duties; on a worldwide, perpetual, irrevocable, royalty-free basis to BMJ Publishing Group Ltd ("BMJ") its licensees and where the relevant Journal is co-owned by BMJ to the co-owners of the Journal, to publish the Work in this journal and any other BMJ products and to exploit all rights, as set out in our licence.

The Submitting Author accepts and understands that any supply made under these terms is made by BMJ to the Submitting Author unless you are acting as an employee on behalf of your employer or a postgraduate student of an affiliated institution which is paying any applicable article publishing charge ("APC") for Open Access articles. Where the Submitting Author wishes to make the Work available on an Open Access basis (and intends to pay the relevant APC), the terms of reuse of such Open Access shall be governed by a Creative Commons licence – details of these licences and which Creative Commons licence will apply to this Work are set out in our licence referred to above.

Other than as permitted in any relevant BMJ Author's Self Archiving Policies, I confirm this Work has not been accepted for publication elsewhere, is not being considered for publication elsewhere and does not duplicate material already published. I confirm all authors consent to publication of this Work and authorise the granting of this licence.

For peer review only

Self-management challenges following hospital discharge for patients with multimorbidity – a longitudinal qualitative study of a motivational interviewing intervention

Carina Brandberg, Doctoral student [1]

Mirjam Ekstedt, PhD [1,2]*

Maria Flink, PhD [3]

[1]Department of Learning, Informatics, Management and Ethics, LIME, Karolinska Institutet, Stockholm, Sweden

[2]Department of Health and Caring Sciences, Linnaeus University, Kalmar, Sweden

[3]Department of Neurobiology, Care Sciences and Society, Karolinska Institutet, Stockholm, Sweden

*Corresponding author:

Mirjam Ekstedt, PhD

Department of Health and Caring Sciences

Hus Vita, Linnaeus University

SE-391 82 Kalmar, Sweden

Mirjam.ekstedt@lnu.se

Phone: +46 480 446 39

For peer review only - http://bmjopen.bmj.com/site/about/guidelines.xhtml

Abstract

Objectives The aim of this study was to describe challenges in self-management activities among people with multimorbidity during a four-week post-discharge period.

Design This is a longitudinal qualitative study using data from a randomized controlled trial (RCT) of motivational interviewing (MI) sessions.

Setting The RCT was conducted at six wards in two hospitals – one university hospital and one general hospital in Stockholm, Sweden during 2016–2018.

Participants Sixteen participants from the intervention group, diagnosed with heart failure or chronic obstructive pulmonary disease and at least one other chronic condition, were purposively selected for this study.

Interventions Each participant had four or five post-discharge MI sessions with a trained social worker during a period of approximately four weeks. The sessions were recorded digitally and analyzed using content analysis. Altogether, 70 recorded sessions were analyzed.

Results Self-management after hospital discharge was a dynamic process with several shifting features that evolved gradually over time. Patients with multimorbidity experienced two major challenges with self-management in the first four weeks following hospital discharge: 'Managing a system-centered care' and 'Handling the burden of living with multiple illnesses at home post-discharge'.

Conclusions Self-management for patients with multimorbidity in the first post- discharge period does not equate to a fixed set of tasks, but varies over the post-discharge period. Self-management challenges include not only the burden of the disease itself, but also that of navigating and understanding the healthcare system. Hence, self-management support post-

For peer review only

discharge involves both aiding patients with care coordination **and** meeting their gradually shifting disease-related needs.

Trial registration number: Trial Registration Number: NCT02823795, date of registration 2016-07-05.

Keyword: Self-management, patient- centered care, multimorbidity, patient transfer, Motivational Interviewing, qualitative research

Strengths and limitations of this study

- The longitudinal design with repeated sessions between patients and social workers ensure large amounts of high-quality data.
- The patients were selected, after the randomization process, to achieve as rich a variety of data as possible, by including different genders, ages, coaches, and hospital settings.
- A small number of participants (n 16); however, saturation was achieved and the repeated sessions included 70 recorded and analyzed sessions.
- All patients came from same geographical area (Stockholm county, Sweden), which is a limitation regarding transferability.

For peer review only - http://bmjopen.bmj.com/site/about/guidelines.xhtml

Introduction

In Sweden, about 25% of the population have multimorbidity, and this group accounts for 50% of the total healthcare costs (1). There are many definitions of multimorbidity (2), but one frequently used in literature – and in this study – is the co-existence of two or more chronic conditions, where all diagnoses can be of equal importance (3, 4). Healthcare systems all around the world are organized around single diseases (4-7) and most clinical practice guidelines focus on the management of a single disease (8). Such fragmentation of services causes a large number of negative consequences, such as medical errors and misdiagnosis (9). It is therefore not surprising that patients with multimorbidity encounter challenges in the healthcare system, or that patients are especially vulnerable during care transitions.

Discharge from hospital to home is a challenging care transition (10), with known obstacles in the form of adverse events (11, 12), re-hospitalization (13), and new or worsening symptoms (14). A crucial factor for effective care transition to home is support of patient self-management, preferably initiated at the hospital; studies indicate a beneficial effect on the risk for 30-day readmission (15, 16). Self-management is "an individual's ability to manage the symptoms, treatment, physical and psychosocial consequences and life style changes inherent in living with a chronic condition" (17). A patient's capacity for self-management seems to be a crucial factor in care transitions, as high levels of patient activation have been associated with decreased healthcare usage (18). Self-management support during care transitions is especially important, as patients often feel that the discharge encounter does not prepare them for post-hospitalization self-management and that no one takes overall responsibility for the coordination of their care (19). There is an international consensus that the healthcare system needs to be reorganized to provide continuity, coordination, and – most importantly – a patient-centered focus that supports self-management (19), adapted to each patient's

For peer review only

For peer review only - http://bmjopen.bmj.com/site/about/guidelines.xhtml

preferences and goals (6). People with multimorbidity are a population in true need of patient-centered self-management support, taking their preferences and needs into account (20).

Challenges to and facilitators of self-management have been extensively explored, but self-management over time is scarcely described (21). There are even fewer descriptions of how challenges to self-management are experienced and handled during the most vulnerable period, i.e., the first weeks after a hospital discharge – to our knowledge there are none. This study aimed to describe the process of and challenges to self-management activities, as expressed by patients with multimorbidity in a 4-week post-discharge motivational interviewing (MI) consultation trial.

Materials and Methods

Study design

This is a longitudinal qualitative study using inductive qualitative content analysis for analysis. The longitudinal approach was used to detect changes over time in patients' self-management challenges. The data consists of descriptions of self-management challenges after hospitalization by patients with multimorbidity, collected in four to five MI sessions per patient in a randomized controlled trial (RCT). For details of the RCT, see the study protocol (22). A pilot study with three participants was performed before the start of the study, to test the MI method.

The consolidated criteria for reporting qualitative research (COREQ) were used for explicit and comprehensive reporting (23).

The MI intervention

The MI is grounded in self-determination theory, a macro-theory of human motivation. In this study, MI was used to increase patient activation in self-management through enhancing a

For peer review only - http://bmjopen.bmj.com/site/about/guidelines.xhtml

person's autonomy, competence, and relatedness (20). When people become more motivated, engaged, and experience more psychological well-being, this predicts positive health behavior (24). Three specific components of autonomy support are suggested: taking the perspective of the patient, providing the patient with choices, and providing a rationale when choices are not possible (25, 26). The principle of autonomy support is at the heart of the clinical approach called MI (27), which can be delivered in a single session or through multiple sessions (28).

The patients in the intervention group each had four to five MI sessions with one of three trained hospital social worker during a period of approximately four weeks. The first session was conducted 1–2 days after discharge, to establish a first contact and capture any questions that arose early post-discharge. The following sessions were conducted 1, 2, 3, and 4 weeks post-hospitalization. After the fourth session some patients found that they managed their situation and wanted to end the coaching. The MI sessions were held either over the phone or face-to-face in clinic, depending on what the patient preferred. The length of the MI sessions varied between 10 and 90 minutes. The first session was usually the shortest, due to fatigue and having a lot of information to absorb post-discharge. All sessions were audio-recorded with the permission of the patients.

All three hospital social workers had MI training and received MI guidance from a psychologist, a member of MINT (Motivational Interviewing Network of Trainers), once a month during the trial. The core of the MI sessions was to motivate and empower the patient to take active part in self-management regarding four important aspects and to create goals for these aspects: 1) managing medications, 2) managing symptoms and/or signs of worsening illness after discharge, 3) acquiring knowledge of follow-up, and 4) acquiring knowledge of and control over whom to contact for different healthcare needs (22). The patients were also encouraged to discuss other aspects of their self-management that were important to them. The hospital social workers had no prior relationship with the patients.

For peer review only - http://bmjopen.bmj.com/site/about/guidelines.xhtml

For peer review only

Selection of participants and data

Recruitment for the MI intervention (22) was conducted at six wards in two hospitals: one university hospital and one general hospital in Stockholm, Sweden. Data were collected from 2016-08-24 to 2018-05-17. The participants were recruited to the RCT during hospitalization for any cause, if they fulfilled the following inclusion criteria: diagnosed with chronic heart failure (CHF) or/and chronic obstructive pulmonary disease (COPD), able to speak and understand enough Swedish to participate in the MI sessions, not diagnosed with dementia or mild cognitive impairment, and not having a non-resuscitate statement in their medical record. Patients received both written and verbal information about the study and the authors, with the possibility to discuss any questions they might have. Those who needed time to decide were offered that until discharge.

From a total of 207 patients in the RCT, 16 patients from the intervention group (n = 104) were purposively selected to cover as many aspects as possible in this heterogeneous group of patients. Among participants with at least two chronic conditions, we sought a variation in type of diagnoses, ages, genders, and hospitalization at different hospital wards. The patients were also selected to cover MI sessions held by all three coaching social workers and throughout the intervention period, to get a variation in coaches experiences. An overview of the patients' characteristics can be found in Table 1. The MI sessions, i.e., conversations between the patients and the social workers, were audio-recorded and transcribed verbatim. Transcribed data consisted of four to five MI sessions per patient: 70 recorded sessions in all. When the sessions of 16 patients had been analyzed, we analyzed two more series of sessions (i.e., from two patients) to explore if we had reached data saturation of themes for individuals (29). As no additional individual themes emerged from this analysis, we concluded that we had reached sufficient data saturation. No participants dropped out.

For peer review only - http://bmjopen.bmj.com/site/about/guidelines.xhtml

Table 1. Characteristics of the participants (N = **16**).

Characteristics	Number (SD)
**Age, mean**	71.0 (10.0)
Male, mean	69.3 (11.0)
Female, mean	72.1 (9.5)
**Gender**	
Female	7
**Place of birth**	
In Sweden	12
**Marital status**	
Married	7
Single/widowed/separated	9
**Closest next-of-kin**	
Relative[1]	14
No next-of-kin	2
**Education level**	
No education	1
Elementary school	5
High school/upper secondary school	7
College/university	3
**Income (Swedish krona/month)**	
< 10,000	1
10,000–20,000	6
20,000–50,000	7
> 50,000	1
**Specific diagnosis[2]**	
Congestive heart failure	9

For peer review only - http://bmjopen.bmj.com/site/about/guidelines.xhtml

Chronic obstructive pulmonary diseases	9
Hypertension	7
Diabetes	6
Renal failure	3
Anemia	3
**Chronic conditions per individual (N)**	
2–4	2
5	6
6	4
7	4
**Diagnoses (N), males, mean**	6.0 (1.0)
**Diagnoses (N), females, mean**	5.0 (1.4)
**Charlson comorbidity index, age-adjusted, mean**	6.7 (2.5)

[1]Husband/wife/partner, child, or friend.

[2]Data taken from electronic/medical records.

Analysis

The analysis was conducted in two phases: a qualitative content analysis of each individuals' MI sessions (30), and a longitudinal analysis of changes in self-management process based on the content analysis (31). Longitudinal analysis is a qualitative research approach that is well-suited to detect changes across time periods.

The transcribed sessions were analyzed in several steps using inductive qualitative content analysis, including open coding and creating categories through abstraction (30). Researchers' reflections were continuously written down during the analysis and these reflections were used in the analysis process. Initially, the full sequence of the four to five recorded MI

For peer review only - http://bmjopen.bmj.com/site/about/guidelines.xhtml

sessions for each patient was listened to, and the transcripts were read as a whole, to achieve a comprehensive understanding of the experience of self-management after discharge. In the first phase of the analysis, units of meaning were selected, condensed, and coded for one participant at a time, starting from the first session and continuing in chronological order. Thereafter, an axial analysis was conducted where codes from all patients in each session separately were abstracted and formulated to sub-categories, i.e., all codes from the first sessions, then all from the second sessions, and so on. The session-specific sub-categories were thereafter sorted into 14 sub-generic categories, and these were abstracted into two generic categories. Finally, a longitudinal analysis was conducted across the five sessions with focus on changes in the self-management process over time (31). This longitudinal analysis identified a main category across the generic categories, 'dynamic self-management process after discharge.' An example of the analytical steps from units of meaning to generic category can be found in table 2.

Table 2. An example of the analytical steps from units of meaning to generic category (ID 16).

Unit of meaning	Heading (Code)	Sub-category	Sub-generic category	Generic category
Mm...and with home care and other help, you´re never really free	Need for home care decreases sense of freedom	Need for independence delays care seeking	Personal experiences direct care seeking behavior	'Handling the burden of living with multiple illnesses post-discharge.'

CB made the first reading and coding of data. CB is doctoral student and received continuous supervision by MF and ME during the study and obtained training within qualitative research. The coding and interpretation processes were conducted in continuous discussions between the three authors (CB, MF, and ME). Excel was used to sort codes and categories. CB and ME

For peer review only - http://bmjopen.bmj.com/site/about/guidelines.xhtml

are registered nurses, and MF is social worker. MF and ME are well experienced in qualitative research.

Patient and public involvement

Patients were not explicitly involved in the design or analysis of this study, but the study was designed based on extensive exploration of literature and own research. However, the used method (MI) in this study is patient-centred, meaning that the topics discussed during the intervention were guided by patients' needs and interests.

Patients were offered to take part of the result and those who are interested will receive the published article including a plain language summary.

Result

Our data showed that patients with multimorbidity struggled with self-management in the first four weeks at home post-discharge. The longitudinal main category identified, 'A dynamic self-management process after discharge,' consisted of two generic categories that characterized this transitional period in a patient's chain of care: 'Managing a system-centered care' and 'Handling the burden of living with multiple illnesses post-discharge.' In the results, the longitudinal main category is first presented followed by the generic categories. The longitudinal main category does not contain any quotations, as it targets the changes in the self-management process over time.

A dynamic self-management process after discharge

The longitudinal analysis showed that self-management after hospital discharge was a dynamic process with several shifting features that evolved gradually over time. Difficulties that appeared during the first week were replaced by new obstacles in the second or third week, i.e., the challenges shifted as the patients recovered and gradually returned to normal

For peer review only - http://bmjopen.bmj.com/site/about/guidelines.xhtml

life. During the first week post-discharge, the first two sessions concerned how the patients adjusted to their changed health conditions and the patients had many questions, especially regarding medication management. Some of the most salient challenges were fatigue, symptoms impairing activities in daily life and causing social isolation, and handling of new medical regimens. The post-discharge period was also characterized by a struggle to understand the new situation, including understanding information from the hospital.

As fatigue gradually declined, a new topic of health-related anxiety and stress was raised during the second week post-discharge (i.e., the third session). For example, anxiety and stress related to feelings of being a burden to relatives, neighbors, or colleagues, being unsure which healthcare contact was responsible for care post-discharge, or not receiving sick pay from the Swedish Social Insurance Agency. Questions regarding management of stress-related problems dominated the following sessions and peaked during the fourth week. A reduction of fatigue and other symptoms impairing daily life could be seen at the last session.

Managing a system-centered care

The patients described the period following hospitalization as not being centered around their needs, but being based on the design of the healthcare system. The patient had to adapt to the system, rather than the system being aligned to their needs as a patient with multimorbidity. As it is now, I have to chase every healthcare professional myself. It isn't the role you should have when you are on sick leave. The whole idea should be that the system takes care of you, not that you should chase the system (ID 105).

Different physicians used different treatment strategies and several of the patients felt that new GPs lacked knowledge about them and their clinical picture. "I have a cardiologist and I have a pulmonologist and I have a general practitioner then, and these three never meet." (ID 20).

For peer review only - http://bmjopen.bmj.com/site/about/guidelines.xhtml

This could result in insufficient information on diagnosis and treatment, when to take medicines, and possible side effects of treatment. "Some doctors do not want to prescribe my medications. Medications that I should have, that I have taken for a long time." (ID 4) Patients in this study also described difficulties with getting in touch with their GPs, sometimes resulting in prolonged improper medical treatment. "… then I have to keep track of this doctor at the healthcare center and talk to him during his phone hours, as he has 30 minutes two days a week and so he has no phone hours today." (ID 25)

High turnover of GPs, shortage of time in consultations, and neglect of the patient perspective contributed to non-trusting relationships between patients and GPs. The high turnover of GPs affected patients' continuity of care. "I have been assigned to him, I have not chosen a doctor myself and everything feels very uncertain. You have to start all over again and that is really hard." (ID 51)

For patients with newly diagnosed heart failure, the follow-up after discharge was conducted at a heart failure reception, where each patient got further information, the possibility to ask questions, and, if needed, evaluation and adjustment of medicines. Despite this, patients lacked an opportunity to discuss their medications with a physician, instead of having a specialist nurse evaluating the physician's prescriptions. In this way, the heart failure reception was perceived to add to the fragmentation through involving yet another healthcare contact. "Yeah, so I met with the nurse at the cardiac ward this past Monday and I don't know, it felt a little out of sync … what the doctors said and what she said, if you can put it that way " (ID 105). In one case, a patient showed creativity by making an Excel file organizing all his 21 healthcare contacts by color, based on what diagnosis they provided care for. During the MI sessions, strategies on how to prepare for healthcare encounters to overcome the lack of continuity were addressed, for example by discussing important topics to mention at meetings.

For peer review only - http://bmjopen.bmj.com/site/about/guidelines.xhtml

Another challenge was that medical record systems differed between departments, forcing patients to take responsibility for gathering information on their own care from their various caregivers. One man said (ID 50): "The healthcare system is made for only one diagnosis at a time." Several participants solved this issue by always carrying their most current patient record and list of medications with them.

Another issue was when the choice of medicine was ruled by price rather than to simplify drug management for the patient. A woman said (ID 69): "They (pharmacy) want to give me Becotide because it is cheaper at the pharmacy, but my heart does not like Becotide and my lung doctor has said 'You must not change it.' And they (pharmacy) say, 'But it is more expensive,' but I'm paying for it." For some patients, the national pharmaceutical policy of offering the cheapest products complicated drug management, as they found it difficult to understand and remember the different generic names. Another barrier regarding medicines was incomplete lists of medicines, especially in the case of temporary medicines, which are often missing from such lists.

Handling the burden of living with multiple illnesses at home post-discharge

Lack of energy and strength in the first weeks after discharge affected the patients' ability to understand information on how to manage new symptoms, and to make the necessary adjustments in daily life. A man said (ID 36): "but I'm a patient, I'm not a normal person, I'm a patient, I'm not functioning A man on new medication post discharge (ID 105): "…should I sort of get more tired than this, then I will not function as a human being ... I plan my activities by… doing something in the morning, because I know that around twelve, I will have to go to bed and sleep 3 hours".

Fatigue and impaired physical functioning also led to the patients feeling socially isolated in their homes. For example, having to take just a few steps on stairs with impaired balance,

For peer review only - http://bmjopen.bmj.com/site/about/guidelines.xhtml

which was a challenge already under ordinary conditions, now significantly affected daily life. In one case, a woman's fear of sudden dizziness and a risk of falling on the stairs led her to repeatedly cancel appointments with her general practitioner (GP). Some of the patients also expressed uncertainty and had a lot of questions when they got home. "…if you have undergone surgery or something at hospital, you receive this note and the doctor rambles on a lot but then, when you get home, you wonder what they actually said." (ID 25)

Self-management also required high level of attentiveness to subtle symptoms in order to identify diagnoses correctly and respond properly. Some patients expressed concerns regarding their ability to identify which disease their symptoms derived from. For example, a patient with COPD, CHF, and anemia (ID 20) misinterpreted her breathing problems as related to her COPD and thus acted on the symptoms "as usual." She said: "Yes, you get used to feeling bad." This delayed her from seeking care, which led to hospitalization for what turned out to be serious anemia. Some patients also expressed difficulties in identifying possible side effects when prescribed several new medicines at once. Some found their own ways of managing their daily medication, for example by delegating this responsibility to relatives.

An acute hospital stay was experienced as a setback, which reduced strength and affected motivation for self-management. It was perceived as having to start all over again, to regain strength and independence in daily life. Keeping physical activity levels up was regarded as especially stressful after a stay in hospital. "I had a walk…or treadmill for a long time, but I haven't been able to walk on it, since I've been in such bad shape, but now… And then there's so much else going on around me and…" (ID 7) Most patients were aware of the importance of physical activity for managing daily life. Challenges to exercise included health limitations, repeated infections, surgery, lack of company, and inclement weather.

For peer review only - http://bmjopen.bmj.com/site/about/guidelines.xhtml

The strong desire for autonomy was an asset in the handling of symptoms and challenges post-discharge. In contrast, adjusting to being dependent on home healthcare or informal care, due to increased fatigue or impaired health, was hard for some. Some participants also described having felt shame since childhood when asking for help, and taking pride in caring for themselves, which was strongly associated with their self-image. One woman said: "I want to take care of myself as well, that's the main thing I do." (ID 51) Such striving for independence could be a health risk due to seeking care too late or refusing treatment such as dialysis because of fears of becoming dependent.

In some cases, a care relationship with a relative or friend may have delayed getting adequate help. In one case, a very attentive neighbor provided food and care for a woman who could not get out of her couch after three serious falls and multiple fractures, which delayed the hospitalization by four weeks. In another case, a patient was cared for at home by her husband for several weeks before she went to hospital, and only then did she realize that she had serious anemia. Lacking the motivation to book an appointment with primary care was also related to having a poor relationship with or lacking trust in the GP; some waited for so long that they had to seek emergency care instead. "What can I say, I'm a little put off by the healthcare center that I've been going to all these years, because they've given me Madopark [levodopa] and antidepressants when I came to them for fatigue and chest tightness in May, so I'm a little like … Yeah, when it comes to my heart, I don't feel that I trust my GP with that anymore. If you have a cold or something, that's different. That isn't life-threatening" (ID 25).

For peer review only - http://bmjopen.bmj.com/site/about/guidelines.xhtml

Discussion

The result of this analysis of a series of MI sessions during four weeks post-hospitalization showed that self-management after discharge is a dynamic process that is affected by managing a system-centered care and the burden of living with multiple illnesses. Overall, patients with multimorbidity need support during the first couple of weeks post-discharge, implying that they are sensitive to a system-centered healthcare system.

The period immediately after discharge from hospital is a vulnerable one, with increased risk of new hospitalizations. Patients with multimorbidity may be especially vulnerable in the management of their chronic illnesses. Our results, in line with previous research (24, 32), showed that self-management is a dynamic process that changes rapidly during the first weeks post-discharge. The patients experienced shifting needs and struggled with self-management in a system that was designed for a single-disease population, not one of increasing age and with an increasing number of diseases. Thus, the patients experienced a triple whammy, as they had to handle the burden of their illness, including fatigue, the burden of self-management of multiple diagnoses, and the burden of being in a system not designed to meet their needs (5-7, 33). In this, the patients experienced that they were left alone to handle their self-management tasks, which especially affected those who were unsure about their own abilities (34). The patients' experiences of a system that did not meet their needs could be reinforced by the design of the Swedish healthcare system, which has one of the shortest lengths of hospital stays in the EU (5.9 days) and the third lowest percentage of general practitioners among countries in the OECD (35). This affects this group of patients, due to a need for prompt follow-up within primary healthcare post-discharge.

The current study also highlights that patients cannot cope with more activities than what is "mandatory" after discharge, as they struggle with self-management while being fatigued. A

For peer review only - http://bmjopen.bmj.com/site/about/guidelines.xhtml
For peer review only

large proportion of those invited to participate in the RCT in this study declined to participate due to fatigue and severe symptoms (36), indicating that patients with complex care needs might need support for more than just self-management during the post discharge period. The system-centered healthcare system was a sub-optimal condition for motivation and self-management of illness post-discharge. The population in this study was in a great need of healthcare that could provide them with a patient- and family-centered approach, as suggested by other studies (3, 37). Initiatives in this direction are seen within a national healthcare reform (38). However, understanding how to provide this type of care efficiently is an enormous challenge (3).

Strengths and limitations

A strength of this study is the longitudinal design with four to five MI sessions, conducted during a four-week post-discharge period. However, there is a need to consider that the audio-recorded MI sessions probably affected the post-discharge period in several ways, as they aimed to increase patients' motivation for self-management. The longitudinal main category "dynamic self-management process after discharge" was considered to capture the patients' transition processes and their experiences of how self-management and recovery changed during the first weeks post-discharge. However, this process might have been different for persons with multimorbidity who did not receive MI sessions.

The MI sessions were directed at four aspects relevant to post-discharge self-management: (1) medication, 2) symptoms, 3) follow-up, and 4) whom to contact for different healthcare needs. However, in accordance with the MI methodology, patients were also encouraged to discuss any relevant problems that occurred post-discharge, meaning that the sessions were guided by patient needs. The results indicated that the patients felt free to discuss several different aspects, both the four mentioned above and others. The MI coaches were hospital

For peer review only - http://bmjopen.bmj.com/site/about/guidelines.xhtml

social workers, i.e., not medically trained staff, which could have led to the sessions focusing on more general aspects of self-management and not specifically on medical aspects.

To ensure trustworthiness (29), researchers' reflections were continuously written down during the analysis. These reflections were used in the analysis process. The analysis was repeatedly discussed between the three authors and within the research group. Examples of quotations from the interviews are presented in the findings. The patients were selected to achieve as rich a variety of data as possible, by including different genders, ages, coaches, and hospital settings. We did not return the results to the participants for verification, as we considered that this group of old, sick patients might not be able to assess the correctness of the analysis (29).

The data collection ensured large amounts of high-quality data, thanks to the repeated sessions between patients and social workers. These sessions contributed to building trust between patients and coaches, which probably made it more likely that rich information was obtained and that any misunderstandings or distortions were uncovered (39).

Conclusion

Self-management for patients with multimorbidity in the first weeks after hospitalization does not equate to a fixed set of tasks, but varies over the post-discharge period. Self-management challenges include not only the burden of the disease itself, but also that of navigating and understanding the healthcare system. Hence, self-management support in the post-discharge period involves both aiding patients with care coordination **and** meeting their gradually shifting disease-related needs.

For peer review only - http://bmjopen.bmj.com/site/about/guidelines.xhtml

Declarations

Ethics approval and consent to participate

The study was approved by the Regional Research Ethics Committee (No. 2014/149831/2) and was performed in line with the Helsinki Declaration (2014). Patients who met the inclusion criteria and agreed to participate provided written informed consent.

Consent for publication

Consent for recording the MI sessions and publication was acquired during the inclusion of the participants.

Availability of data and materials

The datasets used during the current study are available from the corresponding author on request.

Competing interests

The authors declare that they have no competing interests.

Acknowledgements

The authors thank all the patients for their participation in this study. The authors would also like to thank Marita Renman-Bengtcén, social worker, Camilla Lundström, social worker, and Camilla Kjellberg, social worker, for performing all the MI coaching sessions with the patients, and Johan Holmberg, psychologist and member of MINT, and Sara Hammer, psychologist and member of MINT, for mentoring and providing insightful coaching to the coaches.

For peer review only - http://bmjopen.bmj.com/site/about/guidelines.xhtml

Funding

Funding was received from the Doctoral School in Health Care Sciences (FiV) [#4-3084/2014], Karolinska Institutet, FORTE [#2015-00412], and Vårdalstiftelsen [#2014-0026]. The support from FiV and Vårdalsstiftelsen was unconditional. The data collection, design, analysis, interpretation, and reporting were performed without their interference.

Authors' contributions

ME and MF contributed with the conception and design of the study and the obtaining of funding. CB and MF performed inclusion of all participants in the RCT. CB conducted the initial coding of transcribed data in discussion with ME and MF and then performed a longitudinal analysis with a focus on changes in the self-management process over time. In discussions with CB, MF and ME contributed to the abstraction, interpretation of codes, and formulation of sub-categories and main categories. CB drafted the manuscript. MF and ME contributed with substantial contents to the writing. All authors read and approved the final manuscript prior to submission.

For peer review only - http://bmjopen.bmj.com/site/about/guidelines.xhtml

References

1. Vårdanalys M. Vårdval och vårdutnyttjande–Hur fördelas vården mellan olika socioekonomiska grupper vid vårdval i specialistvården. Myndigheten för Vårdanalys, Stockholm[Google Scholar]. 2014.
2. Fratiglioni L, Marengoni A, Meinow B, Karp A. Multipla hälsoproblem bland personer över 60 år - En systematisk litteraturöversikt om förekomst, konsekvenser och vård. Regeringskansliet. Statens Offentliga Utredningar. SOU 2010:48.; 2010.
3. Boyd CM, Fortin M. Future of multimorbidity research: how should understanding of multimorbidity inform health system design?(Report). Public Health Reviews. 2010;32(2):451.
4. Violan C, Foguet-Boreu Q, Flores-Mateo G, Salisbury C, Blom J, Freitag M, et al. Prevalence, Determinants and Patterns of Multimorbidity in Primary Care: A Systematic Review of Observational Studies. PloS one. 2014;9(7):e102149.
5. O'Hare AM, Rodriguez RA, Bowling CB. Caring for patients with kidney disease: shifting the paradigm from evidence- based medicine to patient- centered care. Nephrology Dialysis Transplantation. 2016;31(3):368-75.
6. Salisbury C, Man M-S, Bower P, Guthrie B, Chaplin K, Gaunt DM, et al. Management of multimorbidity using a patient-centred care model: a pragmatic cluster-randomised trial of the 3D approach. The Lancet. 2018;392(10141):41-50.
7. Fratiglioni L, Marengoni A, Meinow B, Karp A. Multipla hälsoproblem bland personer över 60 år.
8. American Geriatrics Society Expert Panel on the Care of Older Adults With M. Patient-Centered Care for Older Adults with Multiple Chronic Conditions: A Stepwise Approach from the American Geriatrics Society. Journal of the American Geriatrics Society. 2012;60(10):1957-68.
9. Kern L, Safford M, Slavin M, Makovkina E, Fudl A, Carrillo J, et al. Patients' and Providers' Views on Causes and Consequences of Healthcare Fragmentation in the Ambulatory Setting: a Qualitative Study. Journal of General Internal Medicine. 2019;34(6):899-907.
10. Organization WH. Transitions of Care: Technical Series on Safer Primary Care 2016. Available from: https://apps.who.int/iris/bitstream/handle/10665/252272/9789241511599-eng.pdf?sequence=1.
11. Forster AJ, Murff HJ, Peterson JF, Gandhi TK, Bates DW. The incidence and severity of adverse events affecting patients after discharge from hospital.(Abstract). Annals of Internal Medicine. 2003;138(3):161.
12. Kripalani S, Lefevre F, Phillips CO, Williams MV, Basaviah P, Baker DW. Deficits in Communication and Information Transfer Between Hospital-Based and Primary Care Physicians: Implications for Patient Safety and Continuity of Care. JAMA. 2007;297(8):831-41.
13. Vashi AA, Fox JP, Carr BG, D'onofrio G, Pines JM, Ross JS, et al. Use of Hospital-Based Acute Care Among Patients Recently Discharged From the Hospital. JAMA. 2013;309(4):364-71.
14. Epstein K, Juarez E, Loya K, Gorman MJ, Singer A. Frequency of new or worsening symptoms in the posthospitalization period. Journal of hospital medicine. 2007;2(2):58.
15. Leppin AL, Gionfriddo MR, Kessler M, Brito JP, Mair FS, Gallacher K, et al. Preventing 30-Day Hospital Readmissions: A Systematic Review and Meta-analysis of Randomized Trials. JAMA Internal Medicine. 2014;174(7):1095-107.
16. Braet A, Weltens C, Sermeus W. Effectiveness of discharge interventions from hospital to home on hospital readmissions: a systematic review. JBI database of systematic reviews and implementation reports. 2016;14(2):106.
17. Barlow J, Wright C, Sheasby J, Turner A, Hainsworth J. Self-management approaches for people with chronic conditions: a review. Patient Education and Counseling. 2002;48(2):177-87.
18. Kinney RL, Lemon SC, Person SD, Pagoto SL, Saczynski JS. The association between patient activation and medication adherence, hospitalization, and emergency room utilization in

For peer review only - http://bmjopen.bmj.com/site/about/guidelines.xhtml

patients with chronic illnesses: A systematic review. Patient Education and Counseling. 2015;98(5):545-52.
19. Flink M, Ekstedt M. Planning for the Discharge, not for Patient Self-Management at Home – An Observational and Interview Study of Hospital Discharge. International Journal of Integrated Care. 2017;17(6).
20. Deci EL, Ryan RM. Self-determination theory in health care and its relations to motivational interviewing: a few comments.(Commentary)(Report). The International Journal of Behavioral Nutrition and Physical Activity. 2012;9:24.
21. Audulv Å. The over time development of chronic illness self-management patterns: a longitudinal qualitative study. BMC public health. 2013;13(1):452.
22. Flink M, Lindblad M, Frykholm O, Kneck Å, Nilsen P, Årestedt K, et al. The Supporting Patient Activation in Transition to Home (sPATH) intervention: a study protocol of a randomised controlled trial using motivational interviewing to decrease re-hospitalisation for patients with COPD or heart failure. BMJ Open. 2017;7(7).
23. Tong A, Sainsbury P, Craig J. Consolidated criteria for reporting qualitative research (COREQ): a 32-item checklist for interviews and focus groups. International journal for quality in health care. 2007;19(6):349-57.
24. Johan YYN, Nikos N, Cecilie T-N, Edward LD, Richard MR, Joan LD, et al. Self-Determination Theory Applied to Health Contexts: A Meta-Analysis. Perspectives on psychological science. 2012;7(4):325-40.
25. Rollnick S. Motiverande samtal i hälso- och sjukvård : att hjälpa människor att ändra beteende. 1. uppl. ed. Miller WR, Butler CC, editors. Lund : Studentlitteratur; 2009.
26. Sheldon KM. Self-determination theory in the clinic: motivating physical and mental health. Williams G, Joiner TE, editors. New Haven, Conn. ; London : Yale University Press; 2003.
27. Miller WR, Rollnick S. Meeting in the middle: motivational interviewing and self-determination theory. The international journal of behavioral nutrition and physical activity. 2012;9(1):25-.
28. Lundahl B, Moleni T, Burke BL, Butters R, Tollefson D, Butler C, et al. Motivational interviewing in medical care settings: A systematic review and meta-analysis of randomized controlled trials. Patient education and counseling. 2013;93(2):157-68.
29. Elo S, Kääriäinen M, Kanste O, Pölkki T, Utriainen K, Kyngäs H. Qualitative Content Analysis: A Focus on Trustworthiness. SAGE Open. 2014;4(1):215824401452263.
30. Elo S, Kyngäs H. The qualitative content analysis process. Journal of Advanced Nursing. 2008;62(1):107-15.
31. Saldana, Johnny. Longitudinal qualitative research:Analyzing change through time. Walnut Creek, CA: Altamira Press; 2003.
32. Ko D, Bratzke LC, Roberts T. Self-management assessment in multiple chronic conditions: A narrative review of literature. International Journal of Nursing Studies. 2018;83:83-90.
33. Clarfield A, Bergman H, Kane R. Fragmentation of care for frail older people--An international problem. Experience from three countries: Israel, Canada, and the United States. Journal of the American Geriatrics Society. 2001;49(12):1714-21.
34. Audulv Å, Norbergh KG, Asplund K, Hörnsten Å. An ongoing process of inner negotiation – a Grounded Theory study of self-management among people living with chronic illness. Journal of Nursing and Healthcare of Chronic Illness. 2009;1(4):283-93.
35. Oecd, European Observatory on Health Systems and P. Sweden: Country Health Profile 2017. Brussels: OECD Publishing; 2017.
36. Flink M, Brandberg C, Ekstedt M. Why patients decline participation in an intervention to reduce re-hospitalization through patient activation: whom are we missing?(Report). Trials. 2019;20(1).
37. Ploeg J, Canesi M, D Fraser K, McAiney C, Kaasalainen S, Markle-Reid M, et al. Experiences of community-dwelling older adults living with multiple chronic conditions: a qualitative study. BMJ Open. 2019;9(3):e023345.

For peer review only - http://bmjopen.bmj.com/site/about/guidelines.xhtml

38. Anna Nergårdh LA, Anna-Klara Brage, Niklas Eklöf, Caroline-Olivia Elgán, Max Printz. God och nära vård-En reform för ett hållbart hälso- och sjukvårdssystem. In: Regeringskansliet, Utredningar SO, editors. 2020. p. 492.
39. Polit DF. Nursing research : generating and assessing evidence for nursing practice. Philadelphia : Wolters Kluwer Health/Lippincott Williams & Wilkins; 2012.

For peer review only

For peer review only - http://bmjopen.bmj.com/site/about/guidelines.xhtml

COREQ (COnsolidated criteria for REporting Qualitative research) Checklist

A checklist of items that should be included in reports of qualitative research. You must report the page number in your manuscript where you consider each of the items listed in this checklist. If you have not included this information, either revise your manuscript accordingly before submitting or note N/A.

Topic	Item No.	Guide Questions/Description	Reported on Page No.
**Domain 1: Research team and reflexivity**			
Personal characteristics			
Interviewer/facilitator	1	Which author/s conducted the interview or focus group?	
Credentials	2	What were the researcher's credentials? E.g. PhD, MD	
Occupation	3	What was their occupation at the time of the study?	
Gender	4	Was the researcher male or female?	
Experience and training	5	What experience or training did the researcher have?	
Relationship with participants			
Relationship established	6	Was a relationship established prior to study commencement?	
Participant knowledge of the interviewer	7	What did the participants know about the researcher? e.g. personal goals, reasons for doing the research	
Interviewer characteristics	8	What characteristics were reported about the inter viewer/facilitator? e.g. Bias, assumptions, reasons and interests in the research topic	
**Domain 2: Study design**			
Theoretical framework			
Methodological orientation and Theory	9	What methodological orientation was stated to underpin the study? e.g. grounded theory, discourse analysis, ethnography, phenomenology, content analysis	
Participant selection			
Sampling	10	How were participants selected? e.g. purposive, convenience, consecutive, snowball	
Method of approach	11	How were participants approached? e.g. face-to-face, telephone, mail, email	
Sample size	12	How many participants were in the study?	
Non-participation	13	How many people refused to participate or dropped out? Reasons?	
Setting			
Setting of data collection	14	Where was the data collected? e.g. home, clinic, workplace	
Presence of non-participants	15	Was anyone else present besides the participants and researchers?	
Description of sample	16	What are the important characteristics of the sample? e.g. demographic data, date	
Data collection			
Interview guide	17	Were questions, prompts, guides provided by the authors? Was it pilot tested?	
Repeat interviews	18	Were repeat inter views carried out? If yes, how many?	
Audio/visual recording	19	Did the research use audio or visual recording to collect the data?	
Field notes	20	Were field notes made during and/or after the inter view or focus group?	
Duration	21	What was the duration of the inter views or focus group?	
Data saturation	22	Was data saturation discussed?	
Transcripts returned	23	Were transcripts returned to participants for comment and/or	

For peer review only

Topic	Item No.	Guide Questions/Description	Reported on Page No.
		correction?	
**Domain 3: analysis and findings**			
Data analysis			
Number of data coders	24	How many data coders coded the data?	
Description of the coding tree	25	Did authors provide a description of the coding tree?	
Derivation of themes	26	Were themes identified in advance or derived from the data?	
Software	27	What software, if applicable, was used to manage the data?	
Participant checking	28	Did participants provide feedback on the findings?	
Reporting			
Quotations presented	29	Were participant quotations presented to illustrate the themes/findings? Was each quotation identified? e.g. participant number	
Data and findings consistent	30	Was there consistency between the data presented and the findings?	
Clarity of major themes	31	Were major themes clearly presented in the findings?	
Clarity of minor themes	32	Is there a description of diverse cases or discussion of minor themes?	

Developed from: Tong A, Sainsbury P, Craig J. Consolidated criteria for reporting qualitative research (COREQ): a 32-item checklist for interviews and focus groups. *International Journal for Quality in Health Care*. 2007. Volume 19, Number 6: pp. 349 – 357

Once you have completed this checklist, please save a copy and upload it as part of your submission. DO NOT include this checklist as part of the main manuscript document. It must be uploaded as a separate file.

For peer review only

BMJ Open

Self-management challenges following hospital discharge for patients with multimorbidity – a longitudinal qualitative study of a motivational interviewing intervention

Journal:	*BMJ Open*
Manuscript ID	bmjopen-2020-046896.R2
Article Type:	Original research
Date Submitted by the Author:	19-Jul-2021
Complete List of Authors:	Brandberg, Carina; Karolinska Institute, LIME<br>Ekstedt, Mirjam; Karolinska Institutet, Dept of Learning, Informatics, Managmenet and Ethics, LIME; Linnaeus University Faculty of Health and Life Sciences, School of Health and Caring Sciences<br>Flink, Maria; Karolinska Institutet, Department of Neurobiology, Care Sciences and Society; Department of Social Work
**Primary Subject Heading**:	Qualitative research
Secondary Subject Heading:	Nursing
Keywords:	Heart failure < CARDIOLOGY, Chronic airways disease < THORACIC MEDICINE, PRIMARY CARE, QUALITATIVE RESEARCH, GENERAL MEDICINE (see Internal Medicine)

SCHOLARONE™
Manuscripts

I, the Submitting Author has the right to grant and does grant on behalf of all authors of the Work (as defined in the below author licence), an exclusive licence and/or a non-exclusive licence for contributions from authors who are: i) UK Crown employees; ii) where BMJ has agreed a CC-BY licence shall apply, and/or iii) in accordance with the terms applicable for US Federal Government officers or employees acting as part of their official duties; on a worldwide, perpetual, irrevocable, royalty-free basis to BMJ Publishing Group Ltd ("BMJ") its licensees and where the relevant Journal is co-owned by BMJ to the co-owners of the Journal, to publish the Work in this journal and any other BMJ products and to exploit all rights, as set out in our licence.

The Submitting Author accepts and understands that any supply made under these terms is made by BMJ to the Submitting Author unless you are acting as an employee on behalf of your employer or a postgraduate student of an affiliated institution which is paying any applicable article publishing charge ("APC") for Open Access articles. Where the Submitting Author wishes to make the Work available on an Open Access basis (and intends to pay the relevant APC), the terms of reuse of such Open Access shall be governed by a Creative Commons licence – details of these licences and which Creative Commons licence will apply to this Work are set out in our licence referred to above.

Other than as permitted in any relevant BMJ Author's Self Archiving Policies, I confirm this Work has not been accepted for publication elsewhere, is not being considered for publication elsewhere and does not duplicate material already published. I confirm all authors consent to publication of this Work and authorise the granting of this licence.

For peer review only

Self-management challenges following hospital discharge for patients with multimorbidity – a longitudinal qualitative study of a motivational interviewing intervention

Carina Brandberg, Doctoral student [1]

Mirjam Ekstedt, PhD [1,2]*

Maria Flink, PhD [3]

[1]Department of Learning, Informatics, Management and Ethics, LIME, Karolinska Institutet, Stockholm, Sweden

[2]Department of Health and Caring Sciences, Linnaeus University, Kalmar, Sweden

[3]Department of Neurobiology, Care Sciences and Society, Karolinska Institutet, Stockholm, Sweden

*Corresponding author:

Mirjam Ekstedt, PhD

Department of Health and Caring Sciences

Hus Vita, Linnaeus University

SE-391 82 Kalmar, Sweden

Mirjam.ekstedt@lnu.se

Phone: +46 480 446 39

For peer review only

For peer review only - http://bmjopen.bmj.com/site/about/guidelines.xhtml

Abstract

Objectives The aim of this study was to describe challenges in self-management activities among people with multimorbidity during a four-week post-discharge period.

Design This is a longitudinal qualitative study using data from a randomized controlled trial (RCT) of motivational interviewing (MI) sessions.

Setting The RCT was conducted at six wards in two hospitals – one university hospital and one general hospital in Stockholm, Sweden during 2016–2018.

Participants Sixteen participants from the intervention group, diagnosed with heart failure or chronic obstructive pulmonary disease and at least one other chronic condition, were purposively selected for this study.

Interventions Each participant had four or five post-discharge MI sessions with a trained social worker during a period of approximately four weeks. The sessions were recorded digitally and analyzed using content analysis. Altogether, 70 recorded sessions were analyzed.

Results Self-management after hospital discharge was a dynamic process with several shifting features that evolved gradually over time. Patients with multimorbidity experienced two major challenges with self-management in the first four weeks following hospital discharge: 'Managing a system-centered care' and 'Handling the burden of living with multiple illnesses at home post-discharge'.

Conclusions Self-management for patients with multimorbidity in the first post- discharge period does not equate to a fixed set of tasks, but varies over the post-discharge period. Self-management challenges include not only the burden of the disease itself, but also that of navigating and understanding the healthcare system. Hence, self-management support post-

For peer review only - http://bmjopen.bmj.com/site/about/guidelines.xhtml

discharge involves both aiding patients with care coordination **and** meeting their gradually shifting disease-related needs.

Trial registration number: Trial Registration Number: NCT02823795, date of registration 2016-07-05.

Keyword: Self-management, patient- centered care, multimorbidity, patient transfer, Motivational Interviewing, qualitative research

Strengths and limitations of this study

- The longitudinal design with repeated sessions between patients and social workers ensure large amounts of high-quality data.
- The patients were selected, after the randomization process, to achieve as rich a variety of data as possible, by including different genders, ages, coaches, and hospital settings.
- A small number of participants (n 16); however, saturation was achieved and the repeated sessions included 70 recorded and analyzed sessions.
- All patients came from same geographical area (Stockholm county, Sweden), which is a limitation regarding transferability.

For peer review only

Introduction

In Sweden, about 25% of the population have multimorbidity, and this group accounts for 50% of the total healthcare costs (1). There are many definitions of multimorbidity (2), but one frequently used in literature – and in this study – is the co-existence of two or more chronic conditions, where all diagnoses can be of equal importance (3, 4). Healthcare systems all around the world are organized around single diseases (4-7) and most clinical practice guidelines focus on the management of a single disease (8). Such fragmentation of services causes a large number of negative consequences, such as medical errors and misdiagnosis (9). It is therefore not surprising that patients with multimorbidity encounter challenges in the healthcare system, or that patients are especially vulnerable during care transitions.

Discharge from hospital to home is a challenging care transition (10), with known obstacles in the form of adverse events (11, 12), re-hospitalization (13), and new or worsening symptoms (14). A crucial factor for effective care transition to home is support of patient self-management, preferably initiated at the hospital; studies indicate a beneficial effect on the risk for 30-day readmission (15, 16). Self-management is "an individual's ability to manage the symptoms, treatment, physical and psychosocial consequences and life style changes inherent in living with a chronic condition" (17). A patient's capacity for self-management seems to be a crucial factor in care transitions, as high levels of patient activation have been associated with decreased healthcare usage (18). Self-management support during care transitions is especially important, as patients often feel that the discharge encounter does not prepare them for post-hospitalization self-management and that no one takes overall responsibility for the coordination of their care (19). There is an international consensus that the healthcare system needs to be reorganized to provide continuity, coordination, and – most importantly – a patient-centered focus that supports self-management (19), adapted to each patient's

For peer review only - http://bmjopen.bmj.com/site/about/guidelines.xhtml

preferences and goals (6). People with multimorbidity are a population in true need of patient-centered self-management support, taking their preferences and needs into account (20).

Challenges to and facilitators of self-management have been extensively explored, but self-management over time is scarcely described (21). There are even fewer descriptions of how challenges to self-management are experienced and handled during the most vulnerable period, i.e., the first weeks after a hospital discharge – to our knowledge there are none. This study aimed to describe the process of and challenges to self-management activities, as expressed by patients with multimorbidity in a 4-week post-discharge motivational interviewing (MI) consultation trial.

Materials and Methods

Study design

This is a longitudinal qualitative study using inductive qualitative content analysis for analysis. The longitudinal approach was used to detect changes over time in patients' self-management challenges (22). The data consists of descriptions of self-management challenges after hospitalization by patients with multimorbidity, collected in four to five MI sessions per patient in a randomized controlled trial (RCT). For details of the RCT, see the study protocol (23). A pilot study with three participants was performed before the start of the study, to test the MI method.

The consolidated criteria for reporting qualitative research (COREQ) were used for explicit and comprehensive reporting (24).

The MI intervention

The MI is grounded in self-determination theory, a macro-theory of human motivation. In this study, MI was used to increase patient activation in self-management through enhancing a

For peer review only - http://bmjopen.bmj.com/site/about/guidelines.xhtml

person's autonomy, competence, and relatedness (20). When people become more motivated, engaged, and experience more psychological well-being, this predicts positive health behavior (25). Three specific components of autonomy support are suggested: taking the perspective of the patient, providing the patient with choices, and providing a rationale when choices are not possible (26, 27). The principle of autonomy support is at the heart of the clinical approach called MI (28), which can be delivered in a single session or through multiple sessions (29).

The patients in the intervention group each had four to five MI sessions with one of three trained hospital social worker during a period of approximately four weeks. The first session was conducted 1–2 days after discharge, to establish a first contact and capture any questions that arose early post-discharge. The following sessions were conducted 1, 2, 3, and 4 weeks post-hospitalization. After the fourth session some patients found that they managed their situation and wanted to end the coaching. The MI sessions were held either over the phone or face-to-face in clinic, depending on what the patient preferred. The length of the MI sessions varied between 10 and 90 minutes. The first session was usually the shortest, due to fatigue and having a lot of information to absorb post-discharge. All sessions were audio-recorded with the permission of the patients. (The manual for the MI-sessions is presented in Appendix 1).

All three hospital social workers had MI training and received MI guidance from a psychologist, a member of MINT (Motivational Interviewing Network of Trainers), once a month during the trial. The core of the MI sessions was to motivate and empower the patient to take active part in self-management regarding four important aspects and to create goals for these aspects: 1) managing medications, 2) managing symptoms and/or signs of worsening illness after discharge, 3) acquiring knowledge of follow-up, and 4) acquiring knowledge of and control over whom to contact for different healthcare needs (23). The patients were also

For peer review only - http://bmjopen.bmj.com/site/about/guidelines.xhtml

For peer review only

encouraged to discuss other aspects of their self-management that were important to them. The hospital social workers had no prior relationship with the patients.

Selection of participants and data

Recruitment for the MI intervention (23) was conducted at six wards in two hospitals: one university hospital and one general hospital in Stockholm, Sweden. Data were collected from 2016-08-24 to 2018-05-17. The participants were recruited to the RCT during hospitalization for any cause, if they fulfilled the following inclusion criteria: diagnosed with chronic heart failure (CHF) or/and chronic obstructive pulmonary disease (COPD), able to speak and understand enough Swedish to participate in the MI sessions, not diagnosed with dementia or mild cognitive impairment, and not having a non-resuscitate statement in their medical record. Patients received both written and verbal information about the study and the authors, with the possibility to discuss any questions they might have. Those who needed time to decide were offered that until discharge.

From a total of 207 patients in the RCT, 16 patients from the intervention group (n = 104) were purposively selected to cover as many aspects as possible in this heterogeneous group of patients. Among participants with at least two chronic conditions, we sought a variation in type of diagnoses, ages, genders, and hospitalization at different hospital wards. The patients were also selected to cover MI sessions held by all three coaching social workers and throughout the intervention period, to get a variation in coaches experiences. An overview of the patients' characteristics can be found in Table 1. The MI sessions, i.e., conversations between the patients and the social workers, were audio-recorded and transcribed verbatim. Transcribed data consisted of four to five MI sessions per patient: 70 recorded sessions in all. When the sessions of 16 patients had been analyzed, we analyzed two more series of sessions (i.e., from two patients) to explore if we had reached data saturation of themes for individuals

For peer review only

For peer review only - http://bmjopen.bmj.com/site/about/guidelines.xhtml

(30). As no additional individual themes emerged from this analysis, we concluded that we had reached sufficient data saturation. No participants dropped out.

Table 1. Characteristics of the participants (N = **16**).

Characteristics	Number (SD)
**Age, mean**	71.0 (10.0)
Male, mean	69.3 (11.0)
Female, mean	72.1 (9.5)
**Gender**	
Female	7
**Place of birth**	
In Sweden	12
**Marital status**	
Married	7
Single/widowed/separated	9
**Closest next-of-kin**	
Relative[1]	14
No next-of-kin	2
**Education level**	
No education	1
Elementary school	5
High school/upper secondary school	7
College/university	3
**Income (Swedish krona/month)**	
< 10,000	1
10,000–20,000	6
20,000–50,000	7
> 50,000	1

For peer review only - http://bmjopen.bmj.com/site/about/guidelines.xhtml

**Specific diagnosis²**	
Congestive heart failure	9
Chronic obstructive pulmonary diseases	9
Hypertension	7
Diabetes	6
Renal failure	3
Anemia	3
**Chronic conditions per individual (N)**	
2–4	2
5	6
6	4
7	4
**Diagnoses (N), males, mean**	6.0 (1.0)
**Diagnoses (N), females, mean**	5.0 (1.4)
**Charlson comorbidity index, age-adjusted, mean**	6.7 (2.5)

¹Husband/wife/partner, child, or friend.

²Data taken from electronic/medical records.

Analysis

The analysis was conducted in two phases: a qualitative content analysis of each individuals' MI sessions (31), and a longitudinal analysis of changes in self-management process based on the content analysis (22). Longitudinal analysis is a qualitative research approach that is well-suited to detect changes across time periods.

The transcribed sessions were analyzed in several steps using inductive qualitative content analysis, including open coding and creating categories through abstraction (31). Researchers'

For peer review only - http://bmjopen.bmj.com/site/about/guidelines.xhtml

reflections were continuously written down during the analysis and these reflections were used in the analysis process. Initially, the full sequence of the four to five recorded MI sessions for each patient was listened to, and the transcripts were read as a whole, to achieve a comprehensive understanding of the experience of self-management after discharge. In the first phase of the analysis, units of meaning were selected, condensed, and coded for one participant at a time, starting from the first session and continuing in chronological order. Thereafter, an axial analysis was conducted where codes from all patients in each session separately were abstracted and formulated to sub-categories, i.e., all codes from the first sessions, then all from the second sessions, and so on. The session-specific sub-categories were thereafter sorted into 14 sub-generic categories, and these were abstracted into two generic categories. Finally, a longitudinal analysis was conducted across the five sessions with focus on changes in the self-management process over time (22). This longitudinal analysis identified a main category across the generic categories, 'dynamic self-management process after discharge.' An example of the analytical steps from units of meaning to generic category can be found in table 2.

Table 2. An example of the analytical steps from units of meaning to generic category (ID 16).

Unit of meaning	Heading (Code)	Sub-category	Sub-generic category	Generic category
Mm…and with home care and other help, you´re never really free	Need for home care decreases sense of freedom	Need for independence delays care seeking	Personal experiences direct care seeking behavior	'Handling the burden of living with multiple illnesses post-discharge.'

CB made the first reading and coding of data. CB is doctoral student and received continuous supervision by MF and ME during the study and obtained training within qualitative research. The coding and interpretation processes were conducted in continuous discussions between

For peer review only - http://bmjopen.bmj.com/site/about/guidelines.xhtml

the three authors (CB, MF, and ME). Excel was used to sort codes and categories. CB and ME are registered nurses, and MF is social worker. MF and ME are well experienced in qualitative research.

Patient and public involvement

Patients were not explicitly involved in the design or analysis of this study, but the study was designed based on extensive exploration of literature and own research. However, the used method (MI) in this study is patient-centred, meaning that the topics discussed during the intervention were guided by patients' needs and interests.

Patients were offered to take part of the result and those who are interested will receive the published article including a plain language summary.

Result

Our data showed that patients with multimorbidity struggled with self-management in the first four weeks at home post-discharge. The longitudinal main category identified, 'A dynamic self-management process after discharge,' consisted of two generic categories that characterized this transitional period in a patient's chain of care: 'Managing a system-centered care' and 'Handling the burden of living with multiple illnesses post-discharge.' In the results, the longitudinal main category is first presented followed by the generic categories. The longitudinal main category does not contain any quotations, as it targets the changes in the self-management process over time.

A dynamic self-management process after discharge

The longitudinal analysis showed that self-management after hospital discharge was a dynamic process with several shifting features that evolved gradually over time. Difficulties that appeared during the first week were replaced by new obstacles in the second or third

For peer review only - http://bmjopen.bmj.com/site/about/guidelines.xhtml

week, i.e., the challenges shifted as the patients recovered and gradually returned to normal life. During the first week post-discharge, the first two sessions concerned how the patients adjusted to their changed health conditions and the patients had many questions, especially regarding medication management. Some of the most salient challenges were fatigue, symptoms impairing activities in daily life and causing social isolation, and handling of new medical regimens. The post-discharge period was also characterized by a struggle to understand the new situation, including understanding information from the hospital.

As fatigue gradually declined, a new topic of health-related anxiety and stress was raised during the second week post-discharge (i.e., the third session). For example, anxiety and stress related to feelings of being a burden to relatives, neighbors, or colleagues, being unsure which healthcare contact was responsible for care post-discharge, or not receiving sick pay from the Swedish Social Insurance Agency. Questions regarding management of stress-related problems dominated the following sessions and peaked during the fourth week. A reduction of fatigue and other symptoms impairing daily life could be seen at the last session.

Managing a system-centered care

The patients described the period following hospitalization as not being centered around their needs, but being based on the design of the healthcare system. The patient had to adapt to the system, rather than the system being aligned to their needs as a patient with multimorbidity.

As it is now, I have to chase every healthcare professional myself. It isn't the role you should have when you are on sick leave. The whole idea should be that the system takes care of you, not that you should chase the system (ID 105).

Different physicians used different treatment strategies and several of the patients felt that new GPs lacked knowledge about them and their clinical picture. "I have a cardiologist and I

For peer review only

have a pulmonologist and I have a general practitioner then, and these three never meet." (ID 20).

This could result in insufficient information on diagnosis and treatment, when to take medicines, and possible side effects of treatment. "Some doctors do not want to prescribe my medications. Medications that I should have, that I have taken for a long time." (ID 4) Patients in this study also described difficulties with getting in touch with their GPs, sometimes resulting in prolonged improper medical treatment. "… then I have to keep track of this doctor at the healthcare center and talk to him during his phone hours, as he has 30 minutes two days a week and so he has no phone hours today." (ID 25)

High turnover of GPs, shortage of time in consultations, and neglect of the patient perspective contributed to non-trusting relationships between patients and GPs. The high turnover of GPs affected patients' continuity of care. "I have been assigned to him, I have not chosen a doctor myself and everything feels very uncertain. You have to start all over again and that is really hard." (ID 51)

For patients with newly diagnosed heart failure, the follow-up after discharge was conducted at a heart failure reception, where each patient got further information, the possibility to ask questions, and, if needed, evaluation and adjustment of medicines. Despite this, patients lacked an opportunity to discuss their medications with a physician, instead of having a specialist nurse evaluating the physician's prescriptions. In this way, the heart failure reception was perceived to add to the fragmentation through involving yet another healthcare contact. "Yeah, so I met with the nurse at the cardiac ward this past Monday and I don't know, it felt a little out of sync … what the doctors said and what she said, if you can put it that way " (ID 105). In one case, a patient showed creativity by making an Excel file organizing all his 21 healthcare contacts by color, based on what diagnosis they provided care

For peer review only - http://bmjopen.bmj.com/site/about/guidelines.xhtml

for. During the MI sessions, strategies on how to prepare for healthcare encounters to overcome the lack of continuity were addressed, for example by discussing important topics to mention at meetings.

Another challenge was that medical record systems differed between departments, forcing patients to take responsibility for gathering information on their own care from their various caregivers. One man said (ID 50): “The healthcare system is made for only one diagnosis at a time.” Several participants solved this issue by always carrying their most current patient record and list of medications with them.

Another issue was when the choice of medicine was ruled by price rather than to simplify drug management for the patient. A woman said (ID 69): “They (pharmacy) want to give me Becotide because it is cheaper at the pharmacy, but my heart does not like Becotide and my lung doctor has said ‘You must not change it.’ And they (pharmacy) say, ‘But it is more expensive,’ but I’m paying for it.” For some patients, the national pharmaceutical policy of offering the cheapest products complicated drug management, as they found it difficult to understand and remember the different generic names. Another barrier regarding medicines was incomplete lists of medicines, especially in the case of temporary medicines, which are often missing from such lists.

Handling the burden of living with multiple illnesses at home post-discharge

Lack of energy and strength in the first weeks after discharge affected the patients’ ability to understand information on how to manage new symptoms, and to make the necessary adjustments in daily life. A man said (ID 36): “but I'm a patient, I'm not a normal person, I’m a patient, I’m not functioning A man on new medication post discharge (ID 105): “…should I sort of get more tired than this, then I will not function as a human being ... I plan my

For peer review only

activities by… doing something in the morning, because I know that around twelve, I will have to go to bed and sleep 3 hours".

Fatigue and impaired physical functioning also led to the patients feeling socially isolated in their homes. For example, having to take just a few steps on stairs with impaired balance, which was a challenge already under ordinary conditions, now significantly affected daily life. In one case, a woman's fear of sudden dizziness and a risk of falling on the stairs led her to repeatedly cancel appointments with her general practitioner (GP). Some of the patients also expressed uncertainty and had a lot of questions when they got home. "…if you have undergone surgery or something at hospital, you receive this note and the doctor rambles on a lot but then, when you get home, you wonder what they actually said." (ID 25)

Self-management also required high level of attentiveness to subtle symptoms in order to identify diagnoses correctly and respond properly. Some patients expressed concerns regarding their ability to identify which disease their symptoms derived from. For example, a patient with COPD, CHF, and anemia (ID 20) misinterpreted her breathing problems as related to her COPD and thus acted on the symptoms "as usual." She said: "Yes, you get used to feeling bad." This delayed her from seeking care, which led to hospitalization for what turned out to be serious anemia. Some patients also expressed difficulties in identifying possible side effects when prescribed several new medicines at once. Some found their own ways of managing their daily medication, for example by delegating this responsibility to relatives.

An acute hospital stay was experienced as a setback, which reduced strength and affected motivation for self-management. It was perceived as having to start all over again, to regain strength and independence in daily life. Keeping physical activity levels up was regarded as especially stressful after a stay in hospital. "I had a walk…or treadmill for a long time, but I

For peer review only - http://bmjopen.bmj.com/site/about/guidelines.xhtml

haven't been able to walk on it, since I've been in such bad shape, but now… And then there's so much else going on around me and…" (ID 7) Most patients were aware of the importance of physical activity for managing daily life. Challenges to exercise included health limitations, repeated infections, surgery, lack of company, and inclement weather.

The strong desire for autonomy was an asset in the handling of symptoms and challenges post-discharge. In contrast, adjusting to being dependent on home healthcare or informal care, due to increased fatigue or impaired health, was hard for some. Some participants also described having felt shame since childhood when asking for help, and taking pride in caring for themselves, which was strongly associated with their self-image. One woman said: "I want to take care of myself as well, that's the main thing I do." (ID 51) Such striving for independence could be a health risk due to seeking care too late or refusing treatment such as dialysis because of fears of becoming dependent.

In some cases, a care relationship with a relative or friend may have delayed getting adequate help. In one case, a very attentive neighbor provided food and care for a woman who could not get out of her couch after three serious falls and multiple fractures, which delayed the hospitalization by four weeks. In another case, a patient was cared for at home by her husband for several weeks before she went to hospital, and only then did she realize that she had serious anemia. Lacking the motivation to book an appointment with primary care was also related to having a poor relationship with or lacking trust in the GP; some waited for so long that they had to seek emergency care instead. "What can I say, I'm a little put off by the healthcare center that I've been going to all these years, because they've given me Madopark [levodopa] and antidepressants when I came to them for fatigue and chest tightness in May, so I'm a little like … Yeah, when it comes to my heart, I don't feel that I trust my GP with that anymore. If you have a cold or something, that's different. That isn't life-threatening" (ID 25).

For peer review only - http://bmjopen.bmj.com/site/about/guidelines.xhtml

Discussion

The result of this analysis of a series of MI sessions during four weeks post-hospitalization showed that self-management after discharge is a dynamic process that is affected by managing a system-centered care and the burden of living with multiple illnesses. Overall, patients with multimorbidity need support during the first couple of weeks post-discharge, implying that they are sensitive to a system-centered healthcare system.

The period immediately after discharge from hospital is a vulnerable one, with increased risk of new hospitalizations. Patients with multimorbidity may be especially vulnerable in the management of their chronic illnesses. Our results, in line with previous research (25, 32), showed that self-management is a dynamic process that changes rapidly during the first weeks post-discharge. The patients experienced shifting needs and struggled with self-management in a system that was designed for a single-disease population, not one of increasing age and with an increasing number of diseases. Thus, the patients experienced a triple whammy, as they had to handle the burden of their illness, including fatigue, the burden of self-management of multiple diagnoses, and the burden of being in a system not designed to meet their needs (5-7, 33). In this, the patients experienced that they were left alone to handle their self-management tasks, which especially affected those who were unsure about their own abilities (34). The patients' experiences of a system that did not meet their needs could be reinforced by the design of the Swedish healthcare system, which has one of the shortest lengths of hospital stays in the EU (5.9 days) and the third lowest percentage of general practitioners among countries in the OECD (35). This affects this group of patients, due to a need for prompt follow-up within primary healthcare post-discharge.

The current study also highlights that patients cannot cope with more activities than what is "mandatory" after discharge, as they struggle with self-management while being fatigued. A large proportion of those invited to participate in the RCT in this study declined to participate

For peer review only - http://bmjopen.bmj.com/site/about/guidelines.xhtml

due to fatigue and severe symptoms (36), indicating that patients with complex care needs might need support for more than just self-management during the post discharge period. The system-centered healthcare system was a sub-optimal condition for motivation and self-management of illness post-discharge. The population in this study was in a great need of healthcare that could provide them with a patient- and family-centered approach, as suggested by other studies (3, 37). Initiatives in this direction are seen within a national healthcare reform (38). However, understanding how to provide this type of care efficiently is an enormous challenge (3).

Strengths and limitations

A strength of this study is the longitudinal design with four to five MI sessions, conducted during a four-week post-discharge period. However, there is a need to consider that the audio-recorded MI sessions probably affected the post-discharge period in several ways, as they aimed to increase patients' motivation for self-management. The longitudinal main category "dynamic self-management process after discharge" was considered to capture the patients' transition processes and their experiences of how self-management and recovery changed during the first weeks post-discharge. However, this process might have been different for persons with multimorbidity who did not receive MI sessions.

The MI sessions were directed at four aspects relevant to post-discharge self-management: (1) medication, 2) symptoms, 3) follow-up, and 4) whom to contact for different healthcare needs. However, in accordance with the MI methodology, patients were also encouraged to discuss any relevant problems that occurred post-discharge, meaning that the sessions were guided by patient needs. The results indicated that the patients felt free to discuss several different aspects, both the four mentioned above and others. The MI coaches were hospital

For peer review only - http://bmjopen.bmj.com/site/about/guidelines.xhtml

social workers, i.e., not medically trained staff, which could have led to the sessions focusing on more general aspects of self-management and not specifically on medical aspects.

To ensure trustworthiness (30), researchers' reflections were continuously written down during the analysis. These reflections were used in the analysis process. The analysis was repeatedly discussed between the three authors and within the research group. Examples of quotations from the interviews are presented in the findings. The patients were selected to achieve as rich a variety of data as possible, by including different genders, ages, coaches, and hospital settings. We did not return the results to the participants for verification, as we considered that this group of old, sick patients might not be able to assess the correctness of the analysis (30).

The data collection ensured large amounts of high-quality data, thanks to the repeated sessions between patients and social workers. These sessions contributed to building trust between patients and coaches, which probably made it more likely that rich information was obtained and that any misunderstandings or distortions were uncovered (39).

Conclusion

Self-management for patients with multimorbidity in the first weeks after hospitalization does not equate to a fixed set of tasks, but varies over the post-discharge period. Self-management challenges include not only the burden of the disease itself, but also that of navigating and understanding the healthcare system. Hence, self-management support in the post-discharge period involves both aiding patients with care coordination **and** meeting their gradually shifting disease-related needs.

For peer review only - http://bmjopen.bmj.com/site/about/guidelines.xhtml

Declarations

Ethics approval and consent to participate

The study was approved by the Regional Research Ethics Committee (No. 2014/149831/2) and was performed in line with the Helsinki Declaration (2014). Patients who met the inclusion criteria and agreed to participate provided written informed consent.

Consent for publication

Consent for recording the MI sessions and publication was acquired during the inclusion of the participants.

Availability of data and materials

The datasets used during the current study are available from the corresponding author on request. The manual for the MI-conversation is provided as supplementary material.

Competing interests

The authors declare that they have no competing interests.

Acknowledgements

The authors thank all the patients for their participation in this study. The authors would also like to thank Marita Renman-Bengtcén, social worker, Camilla Lundström, social worker, and Camilla Kjellberg, social worker, for performing all the MI coaching sessions with the patients, and Johan Holmberg, psychologist and member of MINT, and Sara Hammer, psychologist and member of MINT, for mentoring and providing insightful coaching to the coaches.

For peer review only - http://bmjopen.bmj.com/site/about/guidelines.xhtml

Funding

Funding was received from the Doctoral School in Health Care Sciences (FiV) [#4-3084/2014], Karolinska Institutet, FORTE [#2015-00412], and Vårdalstiftelsen [#2014-0026]. The support from FiV and Vårdalsstiftelsen was unconditional. The data collection, design, analysis, interpretation, and reporting were performed without their interference.

Authors' contributions

ME and MF contributed with the conception and design of the study and the obtaining of funding. CB and MF performed inclusion of all participants in the RCT. CB conducted the initial coding of transcribed data in discussion with ME and MF and then performed a longitudinal analysis with a focus on changes in the self-management process over time. In discussions with CB, MF and ME contributed to the abstraction, interpretation of codes, and formulation of sub-categories and main categories. CB drafted the manuscript. MF and ME contributed with substantial contents to the writing. All authors read and approved the final manuscript prior to submission.

For peer review only

References

1. Vårdanalys M. Vårdval och vårdutnyttjande–Hur fördelas vården mellan olika socioekonomiska grupper vid vårdval i specialistvården. Myndigheten för Vårdanalys, Stockholm[Google Scholar]. 2014.
2. Fratiglioni L, Marengoni A, Meinow B, Karp A. Multipla hälsoproblem bland personer över 60 år - En systematisk litteraturöversikt om förekomst, konsekvenser och vård. Regeringskansliet. Statens Offentliga Utredningar. SOU 2010:48.; 2010.
3. Boyd CM, Fortin M. Future of multimorbidity research: how should understanding of multimorbidity inform health system design?(Report). Public Health Reviews. 2010;32(2):451.
4. Violan C, Foguet-Boreu Q, Flores-Mateo G, Salisbury C, Blom J, Freitag M, et al. Prevalence, Determinants and Patterns of Multimorbidity in Primary Care: A Systematic Review of Observational Studies. PloS one. 2014;9(7):e102149.
5. O'Hare AM, Rodriguez RA, Bowling CB. Caring for patients with kidney disease: shifting the paradigm from evidence- based medicine to patient- centered care. Nephrology Dialysis Transplantation. 2016;31(3):368-75.
6. Salisbury C, Man M-S, Bower P, Guthrie B, Chaplin K, Gaunt DM, et al. Management of multimorbidity using a patient-centred care model: a pragmatic cluster-randomised trial of the 3D approach. The Lancet. 2018;392(10141):41-50.
7. Fratiglioni L, Marengoni A, Meinow B, Karp A. Multipla hälsoproblem bland personer över 60 år.
8. American Geriatrics Society Expert Panel on the Care of Older Adults With M. Patient-Centered Care for Older Adults with Multiple Chronic Conditions: A Stepwise Approach from the American Geriatrics Society. Journal of the American Geriatrics Society. 2012;60(10):1957-68.
9. Kern L, Safford M, Slavin M, Makovkina E, Fudl A, Carrillo J, et al. Patients' and Providers' Views on Causes and Consequences of Healthcare Fragmentation in the Ambulatory Setting: a Qualitative Study. Journal of General Internal Medicine. 2019;34(6):899-907.
10. Organization WH. Transitions of Care: Technical Series on Safer Primary Care 2016. Available from: https://apps.who.int/iris/bitstream/handle/10665/252272/9789241511599-eng.pdf?sequence=1.
11. Forster AJ, Murff HJ, Peterson JF, Gandhi TK, Bates DW. The incidence and severity of adverse events affecting patients after discharge from hospital.(Abstract). Annals of Internal Medicine. 2003;138(3):161.
12. Kripalani S, Lefevre F, Phillips CO, Williams MV, Basaviah P, Baker DW. Deficits in Communication and Information Transfer Between Hospital-Based and Primary Care Physicians: Implications for Patient Safety and Continuity of Care. JAMA. 2007;297(8):831-41.
13. Vashi AA, Fox JP, Carr BG, D'onofrio G, Pines JM, Ross JS, et al. Use of Hospital-Based Acute Care Among Patients Recently Discharged From the Hospital. JAMA. 2013;309(4):364-71.
14. Epstein K, Juarez E, Loya K, Gorman MJ, Singer A. Frequency of new or worsening symptoms in the posthospitalization period. Journal of hospital medicine. 2007;2(2):58.
15. Leppin AL, Gionfriddo MR, Kessler M, Brito JP, Mair FS, Gallacher K, et al. Preventing 30-Day Hospital Readmissions: A Systematic Review and Meta-analysis of Randomized Trials. JAMA Internal Medicine. 2014;174(7):1095-107.
16. Braet A, Weltens C, Sermeus W. Effectiveness of discharge interventions from hospital to home on hospital readmissions: a systematic review. JBI database of systematic reviews and implementation reports. 2016;14(2):106.
17. Barlow J, Wright C, Sheasby J, Turner A, Hainsworth J. Self-management approaches for people with chronic conditions: a review. Patient Education and Counseling. 2002;48(2):177-87.
18. Kinney RL, Lemon SC, Person SD, Pagoto SL, Saczynski JS. The association between patient activation and medication adherence, hospitalization, and emergency room utilization in

For peer review only - http://bmjopen.bmj.com/site/about/guidelines.xhtml

patients with chronic illnesses: A systematic review. Patient Education and Counseling. 2015;98(5):545-52.

19. Flink M, Ekstedt M. Planning for the Discharge, not for Patient Self-Management at Home – An Observational and Interview Study of Hospital Discharge. International Journal of Integrated Care. 2017;17(6).

20. Deci EL, Ryan RM. Self-determination theory in health care and its relations to motivational interviewing: a few comments.(Commentary)(Report). The International Journal of Behavioral Nutrition and Physical Activity. 2012;9:24.

21. Audulv Å. The over time development of chronic illness self-management patterns: a longitudinal qualitative study. BMC public health. 2013;13(1):452.

22. Saldana, Johnny. Longitudinal qualitative research:Analyzing change through time. Walnut Creek, CA: Altamira Press; 2003.

23. Flink M, Lindblad M, Frykholm O, Kneck Å, Nilsen P, Årestedt K, et al. The Supporting Patient Activation in Transition to Home (sPATH) intervention: a study protocol of a randomised controlled trial using motivational interviewing to decrease re-hospitalisation for patients with COPD or heart failure. BMJ Open. 2017;7(7).

24. Tong A, Sainsbury P, Craig J. Consolidated criteria for reporting qualitative research (COREQ): a 32-item checklist for interviews and focus groups. International journal for quality in health care. 2007;19(6):349-57.

25. Johan YYN, Nikos N, Cecilie T-N, Edward LD, Richard MR, Joan LD, et al. Self-Determination Theory Applied to Health Contexts: A Meta-Analysis. Perspectives on psychological science. 2012;7(4):325-40.

26. Rollnick S. Motiverande samtal i hälso- och sjukvård : att hjälpa människor att ändra beteende. 1. uppl. ed. Miller WR, Butler CC, editors. Lund : Studentlitteratur; 2009.

27. Sheldon KM. Self-determination theory in the clinic: motivating physical and mental health. Williams G, Joiner TE, editors. New Haven, Conn. ; London : Yale University Press; 2003.

28. Miller WR, Rollnick S. Meeting in the middle: motivational interviewing and self-determination theory. The international journal of behavioral nutrition and physical activity. 2012;9(1):25-.

29. Lundahl B, Moleni T, Burke BL, Butters R, Tollefson D, Butler C, et al. Motivational interviewing in medical care settings: A systematic review and meta-analysis of randomized controlled trials. Patient education and counseling. 2013;93(2):157-68.

30. Elo S, Kääriäinen M, Kanste O, Pölkki T, Utriainen K, Kyngäs H. Qualitative Content Analysis: A Focus on Trustworthiness. SAGE Open. 2014;4(1):215824401452263.

31. Elo S, Kyngäs H. The qualitative content analysis process. Journal of Advanced Nursing. 2008;62(1):107-15.

32. Ko D, Bratzke LC, Roberts T. Self-management assessment in multiple chronic conditions: A narrative review of literature. International Journal of Nursing Studies. 2018;83:83-90.

33. Clarfield A, Bergman H, Kane R. Fragmentation of care for frail older people--An international problem. Experience from three countries: Israel, Canada, and the United States. Journal of the American Geriatrics Society. 2001;49(12):1714-21.

34. Audulv Å, Norbergh KG, Asplund K, Hörnsten Å. An ongoing process of inner negotiation – a Grounded Theory study of self-management among people living with chronic illness. Journal of Nursing and Healthcare of Chronic Illness. 2009;1(4):283-93.

35. Oecd, European Observatory on Health Systems and P. Sweden: Country Health Profile 2017. Brussels: OECD Publishing; 2017.

36. Flink M, Brandberg C, Ekstedt M. Why patients decline participation in an intervention to reduce re-hospitalization through patient activation: whom are we missing?(Report). Trials. 2019;20(1).

37. Ploeg J, Canesi M, D Fraser K, McAiney C, Kaasalainen S, Markle-Reid M, et al. Experiences of community-dwelling older adults living with multiple chronic conditions: a qualitative study. BMJ Open. 2019;9(3):e023345.

For peer review only - http://bmjopen.bmj.com/site/about/guidelines.xhtml

38. Anna Nergårdh LA, Anna-Klara Brage, Niklas Eklöf, Caroline-Olivia Elgán, Max Printz. God och nära vård-En reform för ett hållbart hälso- och sjukvårdssystem. In: Regeringskansliet, Utredningar SO, editors. 2020. p. 492.
39. Polit DF. Nursing research : generating and assessing evidence for nursing practice. Philadelphia : Wolters Kluwer Health/Lippincott Williams & Wilkins; 2012.

For peer review only

Appendix 1_MANUAL

The intervention consists of four cornerstones to ensure a successful transition from the hospital to the home (medication, care plan/follow-up, symptoms and care contacts). The overall objective of the intervention is that the patient gets a secure transition from hospital to home and becomes aware of how they can be more active in self-care.

Cornerstone	Objective
Medication	The patient has knowledge of and justification for which medications they should take and why, and how they should administer/take the medications.
Care plan/ Follow-up	The patient has knowledge and tools for managing follow-up care and treatment, knows why follow-up should take place, where they can turn in case of any problems, and is motivated to manage their own follow-up/care plan.
Symptoms	The patient has knowledge on expected symptoms and signs of deterioration, so they can seek help at the right time and the right level of care (care contacts)
Care contacts	This cornerstone interconnects with the other three cornerstones, i.e., the matter of the patient's care contacts will be relevant in relation to all of them. The objective is that the patient will have knowledge about their care contacts in specialised care and outpatient care, and a method for easily getting in touch during continued care or follow-up of care.

The setup of the conversations:

1. Introduction: Create security, jointly decide on a topic for the conversation.
2. Investigate the patient's views on the matter and assess motivation.
3. Lead into talk about transformation and try to reinforce self-efficacy: Open questions, adapted based on motivation. Scale/rank questions. Information in dialogue. Investigate any ambivalences. Roll with resistance.
4. Closing: Summarize, try to elicit an undertaking.

Conversation 1. Objective: Check how things have been working since the patient got home and make appointments for future conversations.

Conversations 2–5. Objective: The patient and coach work together to identify problems/dysfunctional habits/preconceptions about the disease/treatment, and set an agenda for which change(s) the patient wants to focus on, identify goals with the change, and motivate the patient to start working towards these goals.

For peer review only

Conversation 1:

I wanted to start with checking how things have worked for you since you got home?

The goal of this project is that the two of us will work together to help you feel that you have control over your disease and how you can manage it at home. If it's okay with you, I'd like us to make an appointment for when we can meet (face-to face or by phone) to talk about that a bit more.

Conversations 2–5:

Would you be willing to start with talking a bit about your disease and how it impacts your life? Can you describe to me what you do at home to feel better?

You are describing a lot of important factors that you feel affect your health and how you can take care of yourself. A few other things that I come to think of as important in taking care of your health are medications, being aware of the various symptoms of your disease, your care plan, and your various care contacts. Would you be willing to talk about any of those areas?

MEDICATIONS

Could you show me which of the medications on your list that you take, and tell me a bit about why and how you take them? What do you usually do to get answers to questions you have about medications? Could you describe to me how you feel it works to take the medications on your medication list exactly as the doctor said? Another thing that I was wondering and wanted to ask you about: what do you usually do to keep track of all your medications?

CARE PLAN/FOLLOW-UP

What was decided in regards to your continued care when you were discharged from hospital? What do you usually do when you're waiting for a follow-up? What do you do to keep track of your appointments and follow-ups before you get a notice?

SYMPTOMS

Can you tell me a bit about how your disease makes itself noticeable? What do you do when you get those symptoms? What do you do when you feel that you are getting worse?

CARE CONTACTS

Could you tell me about which care workers you are usually in touch with and why? What do you do when you want to get in touch? What do you usually do to make sure you get the chance to talk about what matters most to you? What do you usually do to remember what the care workers say?

For peer review only

COREQ (COnsolidated criteria for REporting Qualitative research) Checklist

A checklist of items that should be included in reports of qualitative research. You must report the page number in your manuscript where you consider each of the items listed in this checklist. If you have not included this information, either revise your manuscript accordingly before submitting or note N/A.

Topic	Item No.	Guide Questions/Description	Reported on Page No.
**Domain 1: Research team and reflexivity**			
Personal characteristics			
Interviewer/facilitator	1	Which author/s conducted the interview or focus group?	
Credentials	2	What were the researcher's credentials? E.g. PhD, MD	
Occupation	3	What was their occupation at the time of the study?	
Gender	4	Was the researcher male or female?	
Experience and training	5	What experience or training did the researcher have?	
Relationship with participants			
Relationship established	6	Was a relationship established prior to study commencement?	
Participant knowledge of the interviewer	7	What did the participants know about the researcher? e.g. personal goals, reasons for doing the research	
Interviewer characteristics	8	What characteristics were reported about the inter viewer/facilitator? e.g. Bias, assumptions, reasons and interests in the research topic	
**Domain 2: Study design**			
Theoretical framework			
Methodological orientation and Theory	9	What methodological orientation was stated to underpin the study? e.g. grounded theory, discourse analysis, ethnography, phenomenology, content analysis	
Participant selection			
Sampling	10	How were participants selected? e.g. purposive, convenience, consecutive, snowball	
Method of approach	11	How were participants approached? e.g. face-to-face, telephone, mail, email	
Sample size	12	How many participants were in the study?	
Non-participation	13	How many people refused to participate or dropped out? Reasons?	
Setting			
Setting of data collection	14	Where was the data collected? e.g. home, clinic, workplace	
Presence of non-participants	15	Was anyone else present besides the participants and researchers?	
Description of sample	16	What are the important characteristics of the sample? e.g. demographic data, date	
Data collection			
Interview guide	17	Were questions, prompts, guides provided by the authors? Was it pilot tested?	
Repeat interviews	18	Were repeat inter views carried out? If yes, how many?	
Audio/visual recording	19	Did the research use audio or visual recording to collect the data?	
Field notes	20	Were field notes made during and/or after the inter view or focus group?	
Duration	21	What was the duration of the inter views or focus group?	
Data saturation	22	Was data saturation discussed?	
Transcripts returned	23	Were transcripts returned to participants for comment and/or	

For peer review only

Topic	Item No.	Guide Questions/Description	Reported on Page No.
		correction?	
**Domain 3: analysis and findings**			
Data analysis			
Number of data coders	24	How many data coders coded the data?	
Description of the coding tree	25	Did authors provide a description of the coding tree?	
Derivation of themes	26	Were themes identified in advance or derived from the data?	
Software	27	What software, if applicable, was used to manage the data?	
Participant checking	28	Did participants provide feedback on the findings?	
Reporting			
Quotations presented	29	Were participant quotations presented to illustrate the themes/findings? Was each quotation identified? e.g. participant number	
Data and findings consistent	30	Was there consistency between the data presented and the findings?	
Clarity of major themes	31	Were major themes clearly presented in the findings?	
Clarity of minor themes	32	Is there a description of diverse cases or discussion of minor themes?	

Developed from: Tong A, Sainsbury P, Craig J. Consolidated criteria for reporting qualitative research (COREQ): a 32-item checklist for interviews and focus groups. *International Journal for Quality in Health Care*. 2007. Volume 19, Number 6: pp. 349 – 357

Once you have completed this checklist, please save a copy and upload it as part of your submission. DO NOT include this checklist as part of the main manuscript document. It must be uploaded as a separate file.

For peer review only
